# Coordination through Inhibition: Control of Stabilizing and Updating Circuits in Spatial Orientation Working Memory

**DOI:** 10.1523/ENEURO.0537-20.2021

**Published:** 2021-09-07

**Authors:** Rui Han, Hsuan-Pei Huang, Chia-Lung Chuang, Hung-Hsiu Yen, Wei-Tse Kao, Hui-Yun Chang, Chung-Chuan Lo

**Affiliations:** 1 Institute of Bioinformatics and Structural Biology, National Tsing Hua University, Hsinchu 30013, Taiwan; 2 Institute of Systems Neuroscience, National Tsing Hua University, Hsinchu 30013, Taiwan; 3 Institute of Biotechnology, National Tsing Hua University, Hsinchu 30013, Taiwan; 4 Brain Research Center, National Tsing Hua University, Hsinchu 30013, Taiwan

**Keywords:** Buridan’s paradigm, central complex, *Drosophila melanogaster*, ellipsoid body, spatial orientation memory, working memory

## Abstract

Spatial orientation memory plays a crucial role in animal navigation. Recent studies of tethered *Drosophila melanogaster* (fruit fly) in a virtual reality setting showed that the head direction is encoded in the form of an activity bump, i.e., localized neural activity, in the torus-shaped ellipsoid body (EB). However, how this system is involved in orientation working memory is not well understood. We investigated this question using free moving flies (*D. melanogaster*) in a spatial orientation memory task by manipulating two EB subsystems, C and P circuits, which are hypothesized for stabilizing and updating the activity bump, respectively. To this end, we suppressed or activated two types of inhibitory ring neurons (EIP and P) which innervate EB, and we discovered that manipulating the two inhibitory neuron types produced distinct behavioral deficits, suggesting specific roles of the inhibitory neurons in coordinating the stabilization and updating functions of the EB circuits. We further elucidate the neural mechanisms underlying such control circuits using a connectome-constrained spiking neural network model.

## Visual Abstract

**Figure:**
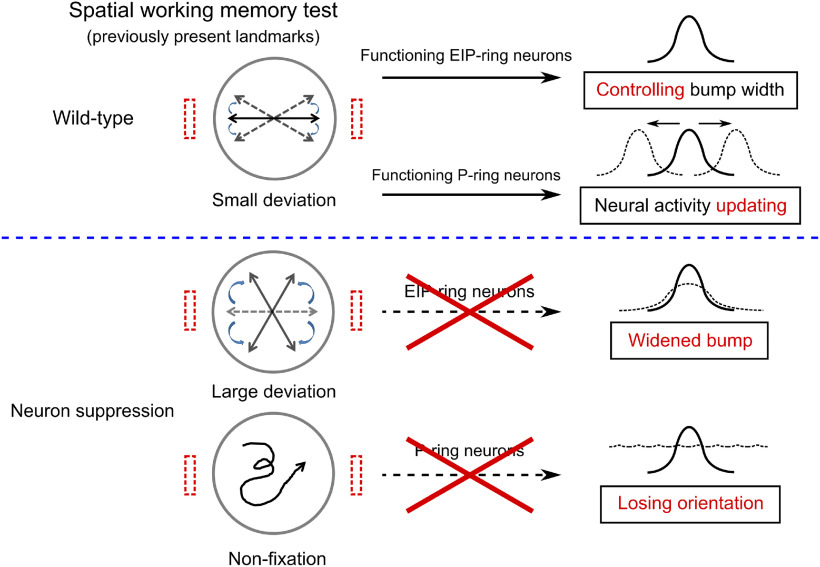

## Significance Statement

Head-direction system has been discovered in rodents for decades. But the detailed neural circuit mechanisms underlying the head-direction system were only described recently by studies of fruit flies on the similar head-direction system. However, how this fruit fly head-direction system involves in orientation memory was not well investigated. The present study addresses this question by investigating free moving flies in a spatial orientation working memory task. By combining neural functional experiments and neural circuit modeling, the study shows how disrupting either of the two subcircuits, one stabilizing and the other updating the neural activity, in the head-direction system leads to different behavioral impairments. The result suggests specific roles of the head-direction subcircuits in the spatial orientation working memory.

## Introduction

Maintaining spatial orientation is a crucial cognitive capability required for animal navigation (Yoder and Taube, 2009; Valerio and Taube, 2016), and understanding the detailed neural mechanisms of spatial orientation is of great interest to researchers in the fields of neurobiology (Hong et al., 2008; Webb and Wystrach, 2016; Webb, 2019) or neuromorphic engineering (Heisenberg and Wolf, 2013; Lin et al., 2013; Robie et al., 2017). In recent years, significant progress has been made in identifying the neural circuits that support spatial orientation (Dewar et al., 2017) in the central complex of *Drosophila melanogaster* (Strauss, 2002; Turner-Evans and Jayaraman, 2016). The central complex has long been associated with short-term spatial memory, visual pattern memory, and motor control (Wolf and Heisenberg, 1991; Liu et al., 2006). The recent discoveries of head-direction selectivity (Muller et al., 1996; Seelig and Jayaraman, 2013, 2015; Fisher et al., 2019; Kim et al., 2019) and localized neural activity in two central complex neuropils, the ellipsoid body (EB) and the protocerebral bridge (PB), have also linked the central complex to the function of spatial orientation (Turner-Evans and Jayaraman, 2016; Dewar et al., 2017). These studies suggested that the head orientation is encoded by localized neural activity, called activity bump, and the bump location in EB shifts in accordance with changes of heading during movement. The function of the EB neurons resemble that of a compass and is, therefore, termed “neural compass” (Clandinin and Giocomo, 2015).

In light of these empirical observations, several neural circuit models of the central complex have been proposed to elucidate the neural circuit mechanisms of head-direction selectivity or other functions associated with the central complex (Cope et al., 2017; Givon et al., 2017; Kim et al., 2017, 2019; Stone et al., 2017; Su et al., 2017; Turner-Evans et al., 2017; Fisher et al., 2019; Pisokas et al., 2020). Some models focused on the stability of the activity bump or on the differences in the circuit dynamics between locus and fruit fly (Kakaria and de Bivort, 2017; Pisokas et al., 2020). Other models studied the plasticity involved in the flexible retinotopic mapping but used simpler firing rate models or schematic models (Fisher et al., 2019; Kim et al., 2019). A large-scale firing-rate neural network model that covered the entire central complex was able to reproduce the steering and homing behavior of bees, but the EB circuits were rather simple with minimal details (Stone et al., 2017).

Recently, a spiking-neuron model of the EB-PB circuits was proposed (Su et al., 2017). The model used a more realistic spiking-neuron model and synaptic dynamics to elucidate how the circuits can maintain a stable activity bump when fruit flies switch between forward movement and rotation states in the absence of landmarks. The model suggested the involvement of two subcircuits: one forms an attractor network and maintains (or stabilizes) an activity bump; the other forms a shifter network and shifts (or updates) the bump position in accordance with changes in body orientation. The model successfully demonstrated the angular errors when a fly moved in darkness (Seelig and Jayaraman, 2015) and predicted the asymmetric activity in the PB during rotation (Green et al., 2017).

The model made an important and unique prediction: the function of spatial orientation working memory requires coordinated activation of the bump-maintaining (or stabilizing) and bump-shifting (or updating) circuits that are controlled by the upstream ring neurons.

However, most of the experimental studies used tethered flies in a virtual reality setting and focused on how manipulation of neurons affects the bump activity. It is not clear how these neurons, in particular those involved in stabilizing and updating the activity bump, play roles in cognition-relevant behavior such as spatial orientation memory in free-moving flies with a more realistic behavioral setting. In the present study, we aimed to address these questions and designed a behavioral task of spatial orientation working memory based on the classic Buridan’s paradigm (Götz, 1980; Strauss and Pichler, 1998; Neuser et al., 2008; Yen et al., 2019; Han et al., 2021). Specifically, we manipulated two types of GABAergic ring neurons (Martín‐Peña et al., 2014) that are hypothesized to control these neurons. Ring neurons project their axons into EB and inhibit neurons including those display the activity bump. Previous studies have reported the roles of the ring neurons in visually-guided behavior (Pan et al., 2009; Ofstad et al., 2011; Thran et al., 2013), ethanol sensitivity (Urizar et al., 2007; Awofala, 2011; Kang et al., 2020), sleep regulation (Donlea et al., 2018; Guo et al., 2018), olfactory memory (Krashes and Waddell, 2008; Zhang et al., 2013) and mating behavior (Becnel et al., 2011; Ishimoto and Kamikouchi, 2020). However, their roles in the working memory of spatial orientation in the presence or absence of visual cues remain unclear. In addition to the neural functional experiments, we also performed computer simulations using the EB-PB model, which produced neural activities that were consistent with the behavioral changes observed in the fruit flies with different experimental conditions. The present study provides a detailed picture on how coordinated activation between the neural processes of stabilization and update plays a crucial role in spatial orientation working memory.

## Materials and Methods

### Fly strains

In the present study we used both male and female flies. Flies were raised at 25°C with a 12/12 h light/dark cycle with a humidity level of ∼50%. Wild-type and transgenic flies were obtained from the Bloomington *Drosophila* Stock Center, *Drosophila* Genomics Resources Center, and Brain Research Center (BRC) in National Tsing Hua University (NTHU). We used wild-type *w^+^* (BRC, NTHU), *;;UAS-Kir2.1* (Guo et al., 2014; Shuai et al., 2015; BRC, NTHU), *;;VT5404-GAL4* (Lin et al., 2013; BRC, NTHU), *;;UAS-CsChrimson.mVenus* (Bloomington stock #55136), *;;UAS-GFP* (Bloomington stock #1522), *tub-GAL80^ts^;;* (Bloomington stock #7017), *c105-GAL4;;* (Humberg et al., 2018; Bloomington stock #30822), *;;UAS-TNT* (Eisel et al., 1993; Sweeney et al., 1995; Bloomington stock #28997), and *;;ninaE* (*Drosophila* Genomics Resources Center #109599). For the neuron suppression experiments, all GAL4 lines were crossed to the *UAS-Kir2.1* or *UAS-TNT* effector lines and the expression can be controlled by temperature with the combination of *tub-GAL80^ts^*. For the optogenetic experiments, all *GAL4* lines were crossed to the *UAS-CsChrimson.mVenus* effector lines. Further information and requests for resources and reagents should be directed to and will be fulfilled by the corresponding author.

### The arena

We conducted the behavioral tasks with flies in a circular arena constructed in house. We followed the general design principles described in a previous study (Yen et al., 2019). The arena used in the present study had a central circular platform of 85 mm in diameter and was surrounded by a 360°

**Figure 1.**
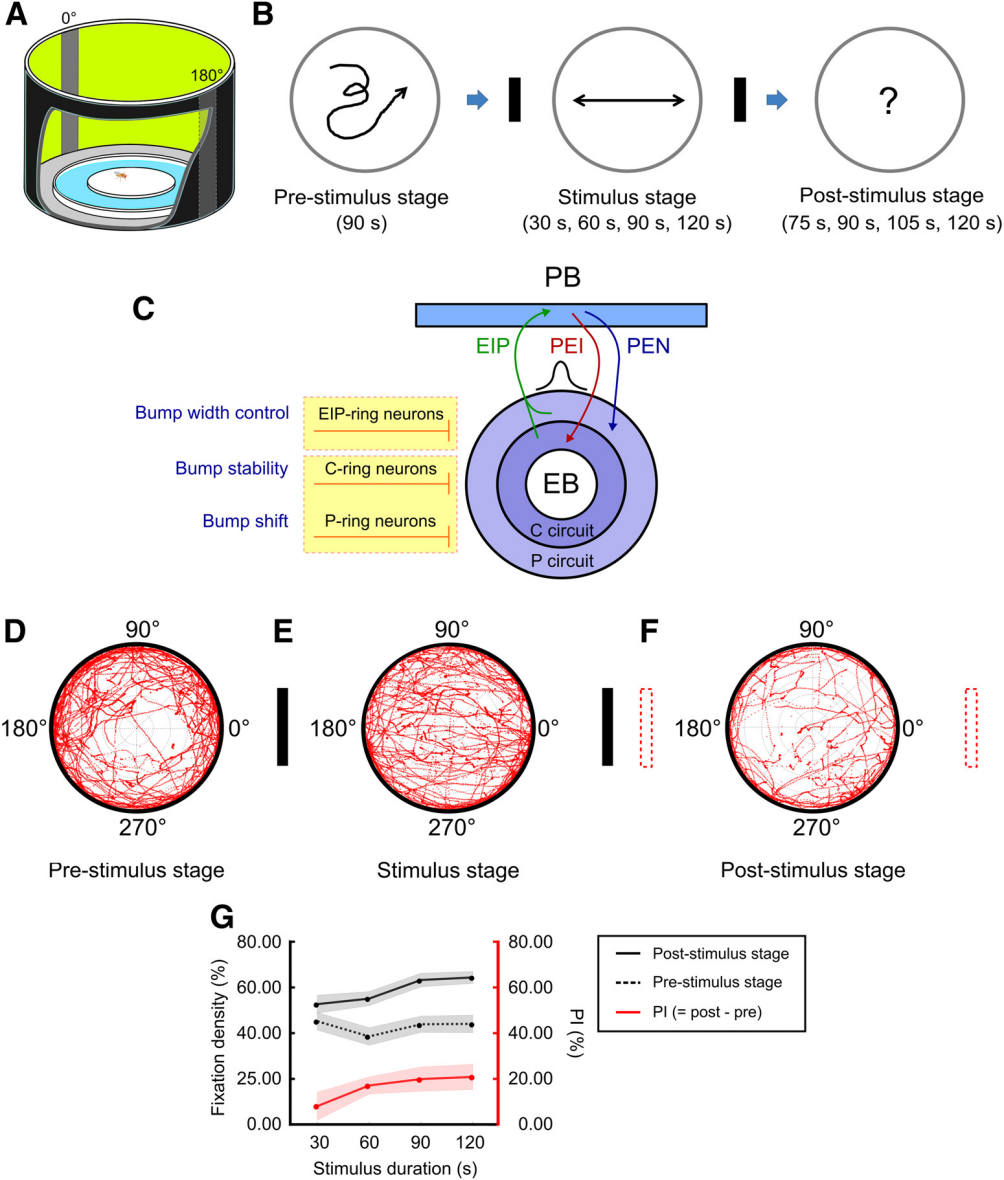
The model and experiment of spatial orientation working memory for *D. melanogaster* (fruit fly). The performance of the wild-type flies indicated strong working memory of the landmark directions. ***A***, The behavior arena featured a central platform surrounded by water and by a 360° LED screen that displayed two vertical black strips as the visual landmarks at 0° and 180°. ***B***, The task, designed based on Buridan’s paradigm (Götz, 1980; Strauss and Pichler, 1998; Neuser et al., 2008), was divided into three stages: the prestimulus stage (90 s), stimulus stage (variable duration), and poststimulus stage (variable duration). In each trial, a single fruit fly was placed at the center of the platform and was allowed to freely move on the platform in the whole trial. ***C***, Schematic diagram of the EB-PB neural network (Su et al., 2017). The EIP, PEI, and PEN neurons support the activity bump which encodes the head direction with respect to a cued location. The bump is modulated by three types of GABAergic ring neurons, EIP-ring, P-ring, and C-ring neurons, and each performs different functions. These ring neurons can be targeted by specific *GAL4* drivers as shown in Extended Data Figure 1-1. ***D–F***, The walking patterns as indicated by the red trajectories of the flies (*n* = 30) in the prestimulus, stimulus, and poststimulus stages. In the stimulus stage, there was a clear pattern of fixation toward the landmarks. The fixation behavior was reduced but still statistically significant in the poststimulus stage. The definition of the movement direction and the trajectory distribution are shown in Extended Data Figure 1-2. ***G***, The fixation density (fixation duration/stage duration) of the poststimulus stage (black solid), the prestimulus stage (black dashed), and their differences, termed *PI* (red) as functions of the stimulus exposure duration. The strength of memory, which is indicated by PI, increases with the stimulus exposure duration. Schematics of neural mechanisms underlying spatial orientation memory and its circuit model are show in Extended Data Figure 1-3. Movement patterns of individual flies are displayed in Extended Data Figure 1-4.

LED display with water filling the basin surrounding the platform (Fig. 1*A*). The display was made of 20 LED panels and each panel consists of four 8 × 8 LED matrices (small 1.2’’ 8 × 8 ultrabright yellow-green LED matrix KWM-30881CUGB). The entire LED screen measured 200 mm in diameter and 130 mm in height. We used green LEDs with a wavelength of 572 nm, which is close to the peak sensitivity of fly eyes (Longden, 2016). Each vertical line of the LEDs could be individually controlled by a personal computer via an Arduino board (Arduino Shield MEGA2560). A CCD camera was mounted directly above the center of the platform and was used to record the movement trajectories of the flies using a Python script developed in house.

10.1523/ENEURO.0537-20.2021.f1-1 Extended Data Figure 1-1 The fluorescent images of the *GAL4* drivers that are expressed in the EIP-ring neurons, P-ring neurons, or C-ring neurons. ***A***, The EIP-ring neurons expressed driver *c105-GAL4*, and the P-ring neurons expressed driver *VT5404-GAL4*. ***B***, The EIP-ring neurons expressed drivers *R31A12-GAL4* (Jenett et al., 2012; the image is from Janelia Research Campus, https://flweb.janelia.org/cgi-bin/view_flew_imagery.cgi?line=R31A12#), *VT039763-GAL4* (Tirian and Dickson, 2017; Janelia Research Campus, https://flweb.janelia.org/cgi-bin/view_flew_imagery.cgi?line=VT039763#), and *VT39763-GAL4*. ***C***, The P-ring neuron expressed control drivers *VT005404-GAL4* (Tirian and Dickson, 2017; Janelia Research Campus, https://flweb.janelia.org/cgi-bin/view_flew_imagery.cgi?line=VT005404#) and *R14G09-GAL4* (Jenett et al., 2012; Janelia Research Campus, https://flweb.janelia.org/cgi-bin/view_flew_imagery.cgi?line=R14G09#). ***D***, The C-ring neuron expressed driver *VT011965-GAL4* (Tirian and Dickson, 2017; Janelia Research Campus, https://flweb.janelia.org/cgi-bin/view_flew_imagery.cgi?line=VT011965#). The Rubin lines *R31A12-GAL4*, *R14G09-GAL4* (Jenett et al., 2012) and the VT lines *VT039763-GAL4*, *VT005404-GAL4*, *VT011965-GAL4* (Tirian and Dickson, 2017) are from FlyLight database (https://www.janelia.org/project-team/flylight) and are provided under CC BY 4.0 license (https://reurl.cc/a5AD29). The drivers shown in ***B*–*D*** are less specific to the target neurons and are also expressed in many other brain regions. Therefore, they are not used in the present study. Download Figure 1-1, TIF file.

**Figure 2.**
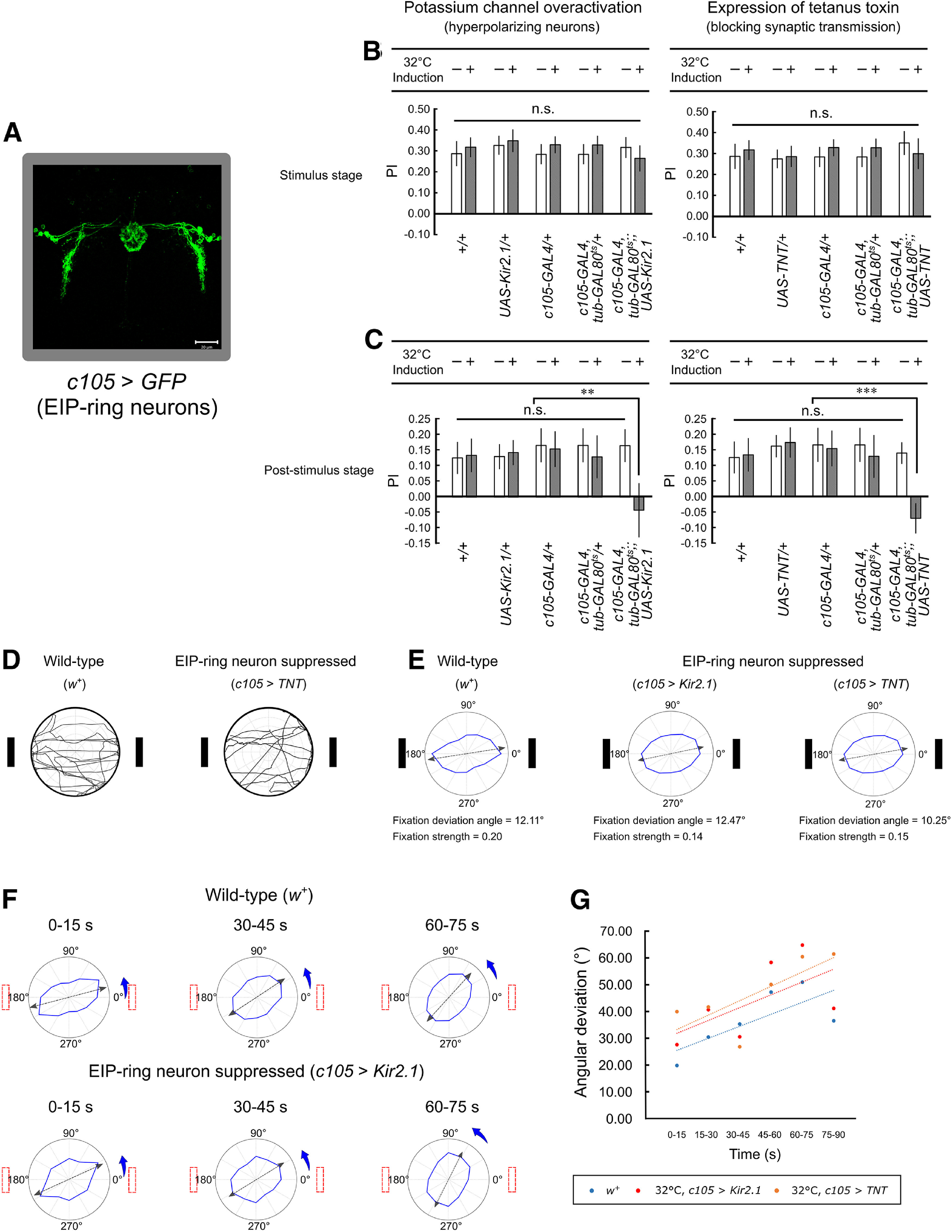
The fruit flies with suppressed EIP-ring neurons exhibited less-accurate orientation memory in the poststimulus stage. ***A***, The *GAL4* expression domains in the EIP-ring neurons (*c105-GAL4;; UAS-GFP*). Eleven virtual slices are used in this confocal image. The thickness of each single slice is 0.90 μm and the thickness of all these 11 slices is 9.88 μm. ***B***, The *PI* of wild-type flies and flies with suppressed EIP-ring neurons (left: 32°C, *c105-GAL4, tub-GAL80^ts^;; UAS-Kir2.1*; right: 32°C, *c105-GAL4, tub-GAL80^ts^;; UAS-TNT*) in the stimulus stage; statistical significance *p < 0.05, **p < 0.01, ***p < 0.001; n.s., non-significant. ***C***, Same as in ***B***, but for the poststimulus stage. ***D***, Example movement trajectories of a control fly (wild type: *w^+^*) and a fly with suppressed EIP-ring neurons (32°C, *c105-GAL4, tub-GAL80^ts^;; UAS-TNT*) during the stimulus stage. ***E***, The radar plots of control flies and flies with suppressed EIP-ring neurons (both methods) in the stimulus stage indicate that the manipulation of EIP-ring neurons did not produce significant effect on fixation behavior. ***F***, Significant effects on the fixation behavior during the poststimulus stage can be observed for flies with suppressed EIP-ring neurons (32°C, *c105-GAL4, tub-GAL80^ts^;; UAS-TNT*) versus the control group (wild type: *w^+^*). The radar plots are displayed for different time windows of the poststimulus stage (Extended Data Fig. 2-1). ***G***, Fixation deviation angle as a function of time for the wild-type files and the flies with EIP-ring neuron suppression during the poststimulus stage. Dashed lines indicate the linear regression of the color-matched data points. The deviation angle was significantly larger in flies with EIP-ring neuron suppression than in the wild-type flies.

10.1523/ENEURO.0537-20.2021.f1-2 Extended Data Figure 1-2 The definition of movement direction and the trajectory distribution. ***A***, In the behavioral task, we recorded the movement direction of the fly in each time step by calculating the vector (black arrow) that connects the positions of the fly in the previous (t1) and present (t2) time steps. The movement direction is defined by the projection (red arrow) of the vector on the LED screen. The movement direction is 356° (or –4°) in this case. ***B***, The population-averaged fixation density (fixation duration/stage duration) toward each pair of quantiles in the prestimulus stage. The fixation density of each pair of quantiles are comparable, suggesting that the flies did not exhibit directional preference in the first stage. ***C***, The distribution of the trajectories of flies on the platform along the radius during the prestimulus stage. The shaded area indicates the SEM. The values were normalized by the area spanned in each unit length of the radius. Download Figure 1-2, TIF file.

**Figure 3.**
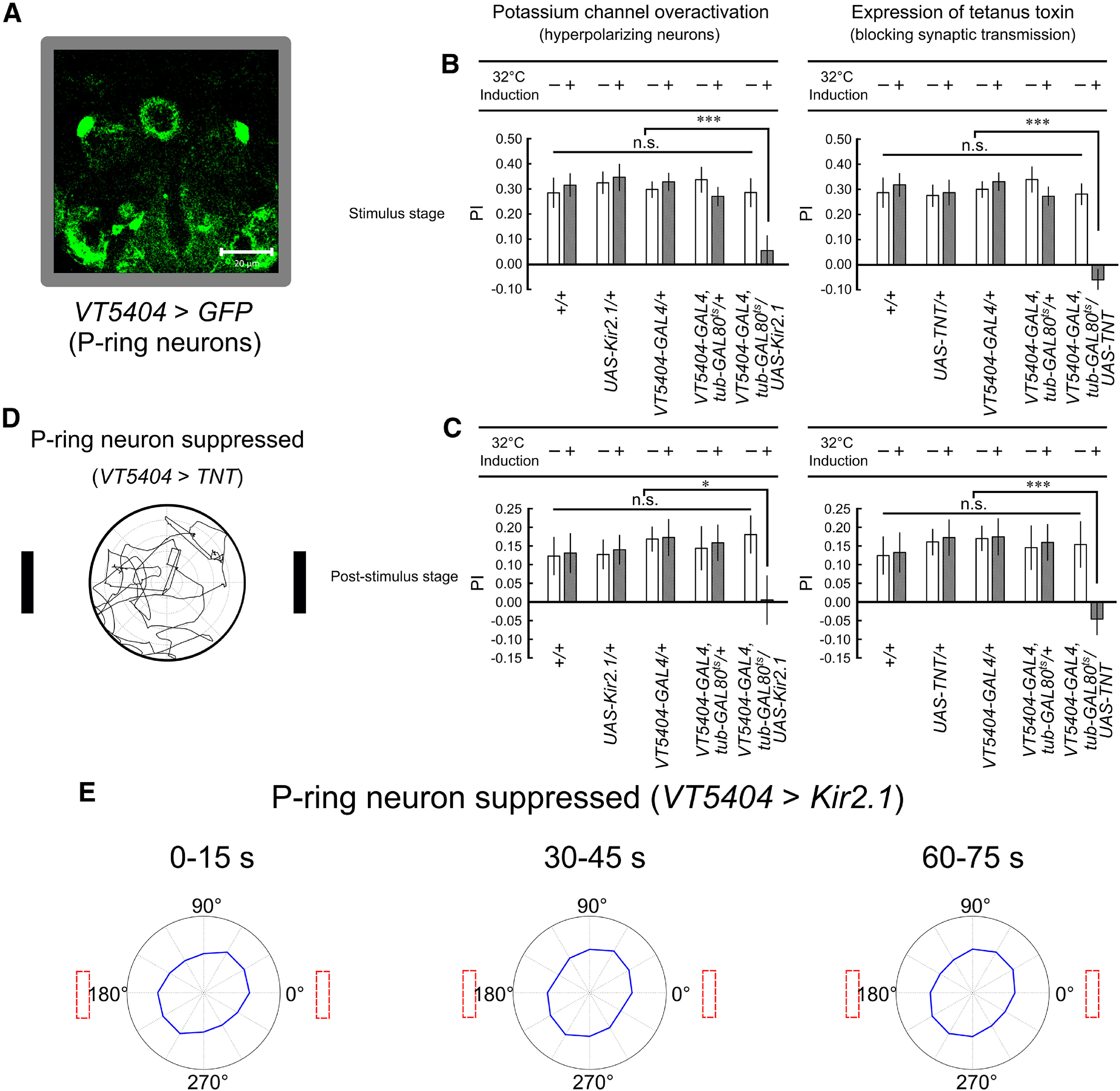
The fruit flies with suppressed P-ring neurons exhibited non-fixation behavior in both stimulus and poststimulus stages. ***A***, *GAL4* expression domains in the P-ring neurons (*;;VT5404-GAL4/UAS-GFP*). Nine virtual slices are used in this confocal image. The thickness of each single slice is 2.06 μm and the thickness of all these nine slices is 18.52 μm. ***B***, The *PI* of the control flies and flies with suppressed P-ring neurons (32°C, *;;VT5404-GAL4, tub-GAL80^ts^/UAS-Kir2.1* and 32°C, *;;VT5404-GAL4, tub-GAL80^ts^/UAS-TNT*) in the stimulus stage. ***C***, Same as in ***B***, but for the poststimulus stage. ***D***, Example movement trajectories of a fly with suppressed P-ring neurons (32°C, *;;VT5404-GAL4, tub-GAL80^ts^/UAS-TNT*) in the stimulus stage. ***E***, Radar plots of flies with suppressed P-ring neurons (32°C, *;;VT5404-GAL4, tub-GAL80^ts^/UAS-Kir2.1*) in different time windows of the poststimulus stage (Extended Data Fig. 3-1); statistical significance *p < 0.05, **p < 0.01, ***p < 0.001; n.s., non-significant.

10.1523/ENEURO.0537-20.2021.f1-3 Extended Data Figure 1-3 Schematics of the fruit fly compass circuits and its model. ***A,*** Fruit fly EB (the grey torus) exhibits localized neural activity, termed activity bump (green), which corresponds to the direction of the visual cue on the screen. **B,** The activity bump persists when the visual cue is turned off. **C,** When the fruit fly changes its heading, the location of the activity bump shifts (or updates) so that it always indicates the head direction with respect to the cued direction. ***D***, The activity bump can be simply generated by localized input to neurons in EB. ***E***, However, according to the attractor network model, the persistency of the bump after offset of the visual cue requires two types of synaptic connections. First, locally recurrent excitation (green and red), which creates the reverberatory activity in the absence of input. Second, the global feedback inhibition (orange), which controls the width of the activity bump so that it does not spread throughout the whole population of neurons. We call these two sets of connections “stabilization circuit.” ***F***, Moreover, the shift of bump location due to the change of heading can be achieved by a shifter (or updater) circuit which form counterclockwise (blue, as shown) or clockwise (not shown) feedforward excitation, which propagates the activity in either direction. ***G***, ***H***, The specific connections as described in panels ***E***, ***F*** were discovered in neurons that interconnect EB and PB in recent connectomic studies. The recurrent excitatory circuits are formed by the EIP (green) and PEI (red) neurons (C circuit), while the shifter circuits are formed by the EIP and PEN (blue) neurons (P circuit). These neurons are modulated by several types of GABAergic ring neurons (orange). The Su et al. (2017) model suggested that the coordinated activation of the C and P circuits are crucial for the function of spatial orientation. ***I***, To simplify the visualization of the circuits, we redraw them using a three-ring representation, which highlights the roles of the ring neurons. The C-ring and P-ring neurons switch on/off the stabilization and shifter circuits, respectively. The EIP-ring neurons are responsible for controlling the width of the activity bump (the global inhibition in the attractor network). This representation is used in the subsequent figures in this paper. ***A–C***, ***G***, ***H*** are adapted from Su et al. (2017). Download Figure 1-3, TIF file.

**Figure 4.**
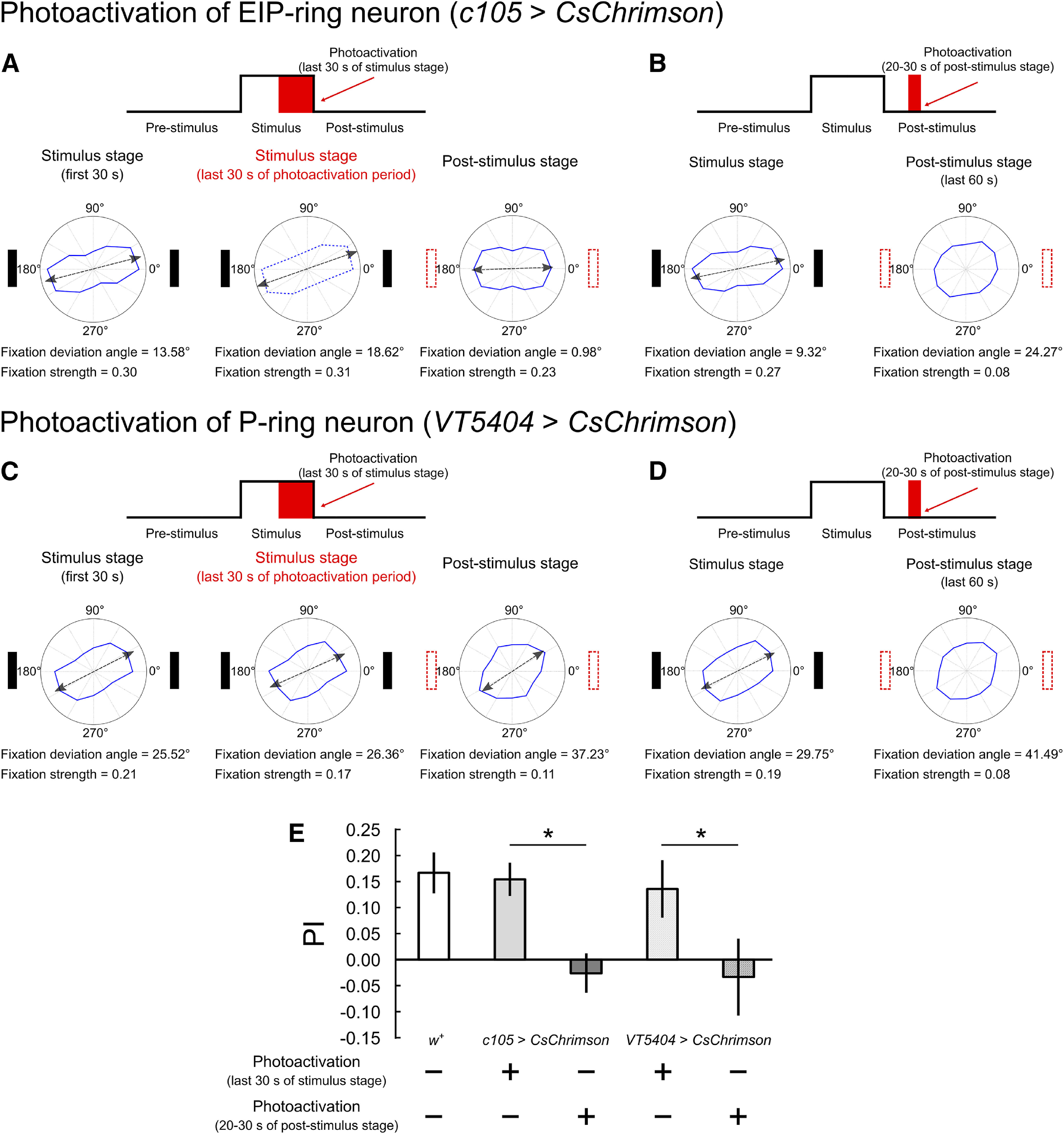
Impact of transient photoactivation of the EIP-ring or P-ring neurons on the spatial orientation working memory. ***A***, Photoactivation of the EIP-ring neurons (*c105-GAL4;;UAS-CsChrimson.mVenus*) during the last 30 s of the stimulus stage. Top, The stimulation protocol. Bottom, The radar plots for the first 30 s of the stimulus stage (left bottom), the last 30 s of the stimulus stage where the photoactivation was applied (middle bottom) and the poststimulus stage (right bottom) indicate that the orientation memory was intact. Note that the flies performed very little movement during the period of the photoactivation (Table 4). ***B***, Same as in ***A***, but with photoactivation during 20–30 s of the poststimulus stage. The radar plots are shown for the stimulus stage and the last 60 s of the poststimulus stage. The fixation behavior was abolished after the photoactivation. ***C***, ***D***, Same as in ***A***, ***B***, but with photoactivation of the P-ring neurons (*;;VT5404-GAL4/UAS-CsChrimson.mVenus*). The photoactivation during the poststimulus stage also abolished the fixation behavior. ***E***, The PI of wild-type flies (*w^+^*) and flies in each photoactivation condition. Photoactivation in the stimulus stage caused minor or no impact on the orientation memory while photoactivation in the poststimulus stage abolished the memory. Performance of control groups (no photoactivation) are shown in Extended Data Figures 4-1, 4-2, 4-3.

10.1523/ENEURO.0537-20.2021.f1-4 Extended Data Figure 1-4 The movement patterns of Individual flies for difference stimulus stage durations. ***A***, Percentage of fixation for four example trials of wild-type flies. The shaded regions indicate the periods in which the percentage of movement toward the landmark positions is more than 16.67% (see Materials and Methods). ***B***, Distribution of the fixation bouts (black bars) in the poststimulus stage for each wild-type flies (stimulus stage duration = 30 s). In this condition, the average fixation duration of each single trial is 39.35 s. ***C***, ***D***, Same in ***A***, ***B***, but with a stimulus duration of 60 s. In this condition, the average fixation duration of each single trial is 49.41 s. ***E***, ***F***, Same in ***A***, ***B***, but with a stimulus duration of 90 s. In this condition, the average fixation duration of each single trial is 66.25 s. ***G***, ***H***, Same in ***A***, ***B***, but with a stimulus duration of 120 s. In this condition, the average fixation duration of each single trial is 77.06 s. Download Figure 1-4, TIF file.

### The *GAL4* drivers

We used *c105-GAL4* line to target the EIP-ring neurons and *VT5404-GAL4* to target the P-ring neurons in the present study (Jenett et al., 2012; Extended Data Fig. 1-1*A*). We have also inspected other EIP-ring neuron expressed drivers, including *R31A12-GAL4* (Omoto et al., 2018), *VT039763-GAL4*, and *VT39763-GAL4* (Lin et al., 2013; Extended Data Fig. 1-1*B*). For P-ring neuron expressed drivers, we have inspected *VT005404-GAL4* and *R14G09-GAL4* (Omoto et al., 2018; Tirian and Dickson, 2017; Extended Data Fig. 1-1*C*). C-ring neuron expressed driver *VT011965-GAL4* (Omoto et al., 2018) are inspected as well (Extended Data Fig. 1-1*D*). However, these drivers are less specific and are expressed in many other brain regions. Specificity of the drivers is crucial to the present study as it involves behavioral experiments. Therefore, we only used *c105-GAL4* and *VT5404-GAL4* in the present study.

### The spatial orientation memory task

We used 3- to 5-d-old flies and clipped the wings 1 d before the experiments (Neuser et al., 2008). In addition to wild-type flies (genotype: *w^+^*), we used flies with suppressed EIP-ring or P-ring neurons, which was achieved by hyperpolarizing the neurons (32°C, *c105-GAL4, tub-GAL80^ts^;; UAS-Kir2.1* for EIP-ring neuron suppression and *;;VT5404-GAL4, tub-GAL80^ts^/UAS-Kir2.1* for P-ring neuron suppression) or by blocking the neurotransmitters (32°C, *c105-GAL4, tub-GAL80^ts^;; UAS-TNT* for EIP-ring neuron suppression and *;;VT5404-GAL4, tub-GAL80^ts^/UAS-TNT* for P-ring neuron suppression).

After the wings were clipped, the flies were placed in an 18 °C incubator in order for *GAL80^ts^* to bind *GAL4* and inhibited the transcription activity of the *GAL4* (control groups), or in a 32°C incubator to relieve the inhibition of *GAL4* proteins so that they can drive the expression of *UAS* (experimental groups; KaiXia et al., 2016) for 1 d. All experiments were performed between 10 A.M. and 5 P.M. We also used optogenetics to transiently activate EIP-ring or P-ring neurons. The expression of the effector (*UAS-CsChrimson.mVenus*) was controlled by red lights (625 nm) exposure after feeding all-*trans*-retinal (100 μm) in a dark environment for 7 d (Wu et al., 2014).

Based on the protocol proposed in an earlier study (Yen et al., 2019), the spatial orientation memory task consisted of three stages: the prestimulus stage, stimulus stage, and poststimulus stage (Fig. 1*B*). Only one fly was submitted to the task in each trial and the fly was allowed to freely move on the circular platform. The prestimulus stage lasted 90 s, during which all LEDs were turned on and no visual landmark was presented on the screen. During the stimulus stage, two vertical black strips, each 30° wide and separated by 180°, were presented on the screen as visual landmarks. We tested four different durations (30, 60, 90, and 120 s) for the stimulus stage with wild-type flies and used 60 s in the subsequent neural functional experiments. In the poststimulus stage, the two landmarks were removed and all LEDs were turned on; this stimulus condition was identical to that used in the prestimulus stage. The duration of the poststimulus stage was 75, 90, 105, and 120 s for the four different durations, 30, 60, 90, and 120 s, of the stimulus stage, respectively. The movement trajectories of the flies were recorded using a CCD camera at a speed of 20–25 frames per second. The position of the fly in each frame was captured by a Python script and saved for postexperiment analysis.

### Optogenetic activation

Red LED array made up of 96 LEDs (625 nm, RED LED SMD 5050) were used to activate *CsChrimson* during the behavioral task. The LED array was placed below the platform in the arena. The platform was made of acrylonitrile-butadiene-styrene (ABS) and was partially transparent to light because of its 1 mm of thickness. We used two protocols of photograph activation. In the first protocol, the red light was turned on during the last 30 s of the stimulus stage. In the second protocol, the red light was turned on for only 10 s starting at 20 s after the poststimulus stage began.

### Confocal images

Seen-day-old adult flies brains were fixed, mounted in PBS as previously described (Chang and Ready, 2000). Images were scanned with Zeiss LSM 510 confocal microscopy.

### Data analysis

One of the goals of the present study was to investigate the alternation of spatial orientation memory, as indicated by the movement direction, under various neural manipulations. Therefore, we designed behavioral measures and analytical methods for this purpose. We first defined the performance index (*PI*) that quantifies how well the flies fixed on the cued locations against a reference direction, i.e., the perpendicular direction. This single-valued measure is easy to be analyzed and compared statistically. However, we discovered that in some conditions, the flies exhibited strong fixation behavior but on the wrong directions. This behavior has a different implication from that exhibiting no fixation. *PI* cannot distinguish the two different behaviors and therefore we designed the radar plot to capture the differences. We present the two analyses for all spatial orientation tasks conducted in the present study and the detailed definitions of the two measures are described below.

To analyze the movement direction of each fly (Liske, 1977) in each video frame, we first calculated the speed vector based on the difference in the coordinates of the fly between two consecutive frames. The movement direction was represented by the degree value, *θ*, on the screen at the point that the speed vector projected to (Extended Data Fig. 1-2). For better presentation and analysis, we calculated the percentages, p(θ), of the movement direction in each of the 12 quantiles in 360°. *θ* represented the degree corresponding to the center of the quantile. Each quantile spanned 30° on the screen. p(θ) was calculated every 5 s with a 15-s sliding time window. Fixation behavior was characterized by a significantly higher percentage of the movement direction falling within ±15° of 0° and 180° on the screen, the quantiles that the two visual landmarks occupied.

To further quantify the performance of the flies in terms of the spatial orientation memory, we defined the *PI*. We first calculated P(0°;180°)=p(0°)+p(180°)

in each stage for each fly. Next, for a given stage, we calculated the fixation density FD(0°;180°), which was defined as the number of 5-s epochs in which P(0°;180°) > 1/6 (or 16.67%) in a stage divided by the total number of epochs of that stage; 16.67% was the expected percentage that a fly spent in two quantiles, e.g., 0° and 180°, if it moved randomly. Because of the highly variable nature of the fly movement, P could be larger than 16.67% in multiple different directions in an epoch. This led to a non-zero FD in more than one direction, even if the fly performed the random walk. Therefore, we stress that measuring FD along in the third stage did not provide correct information about the presence of memory, which should be evaluated by calculating the difference in FD(0°;180°) between the third and the first stage. This is given by *PI* which was defined as

(1) PI=FD(0°;180°)of the 3rd stage−FD(0°;180°)of the 1st stage.

In addition to *PI*, which only measured the fixation behavior at the 0° and 180°, we also visualized the fixation behavior at all directions by the radar plots, which indicated the frequencies p(θ) of the movement direction in each quantile. In the plot, each pair of quantiles that were 180° apart, e.g., quantiles centered at 90° and −90°, was represented by the same value, which was their averaged percentage. This made the radar plot point-asymmetric. If a fly did not exhibit any fixation behavior, it would move toward any direction with equal probability because of the point symmetric property of the arena, and the expected value of each pair of quantiles on the plot was 1/6 (∼16.67%). A value that was significantly different from 1/6 indicates some form of fixation behavior.

The radar plots allowed us to visually inspect the existence of fixation behavior and the main direction that the flies fixated on with respect to the directions of the visual landmarks. To further quantify these two behavioral properties, we calculated the fixation deviation angle ( $\sigma_{f}$) and the fixation strength ( F). The fixation deviation angle ( $\sigma_{f}$) represented the direction of the strongest fixation tendency and was defined by the angle σ, which yielded the smallest second moments M(σ) on the radar plot:

(2) $$M(\sigma_{f})\equiv \min_{\sigma }\left(\sum_{i}p{\left(\theta_{i}\right)}^{2}{\text{sin}}^{2}\left(\theta_{i}-\sigma \right)\right),$$

where $\theta_{i}$ is the angle of each quantile (0°, 30°, 60°, 90°, ….). $\sigma_{f}$ can be obtained by taking the derivative of M(σ) with respect to σ.

(3) $$\frac{dM(\sigma )}{d\sigma }{|}_{\sigma =\sigma_{f}}=0.$$

Plugging $\theta_{i}$ and $p(\theta_{i})$ into this equation and solving for σ using trigonometric identities, we obtain

(4) $$\sin(2\sigma_{f}+\phi )=0$$

and

(5) $$\sigma_{f}=\frac{-\phi }{2}\text{or}\sigma_{f}=\frac{180-\phi }{2},$$

where ϕ is a variable depending on $p(\theta_{i})$ based on the following equations:

(6) cosϕ=A/Kand sinϕ=B/K

(7) $$A=2p{\left(0{}^{\circ}\right)}^{2}+p{\left(30{}^{\circ}\right)}^{2}-p{\left(60{}^{\circ}\right)}^{2}-2p{\left(90{}^{\circ}\right)}^{2}+p{\left(-30{}^{\circ}\right)}^{2}-p{\left(-60{}^{\circ}\right)}^{2}$$

(8) $$B=\sqrt{3}\left(p{\left(-30{}^{\circ}\right)}^{2}-p{\left(30{}^{\circ}\right)}^{2}+p{\left(-60{}^{\circ}\right)}^{2}-p{\left(60{}^{\circ}\right)}^{2}\right)$$

(9) $$K=\sqrt{A^{2}+B^{2}}.$$

The deviation angle given in Equation 6 corresponds to two values. One gives rise to the minimum value of M (denoted as $M_{min}$) and the other gives rise to the maximum value of M (denoted as $M_{max}$). Finally, the fixation strength (*FS*) is defined as follows:

(10) $$FS=1-\frac{M_{min}}{M_{max}}.$$

To determine what level of *FS* indicates a significant fixation, we analyzed the *FS* for all flies in the control groups, during the prestimulus stage. The average *FS* was 0.0544, and the standard deviation was 0.0273. We took two standard deviations above the mean (*FS* = 0.1090) as our statistical criterion for the fixation behavior.

We used the *PI* and the *FS* to quantify the fixation behavior. Although they seem to be redundant, they serve different purposes. The *PI* specifically measures the difference in the fixation (on the cued directions) between two stages and is an indicator of the spatial orientation memory if *PI* is measured for the third and first stages. *PI* does not measure whether the flies also fixate on the other directions. By contrast, the *FS* measures the fixation toward any direction. The exact azimuth angle of the fixation is specified by $\sigma_{f}$. *FS* is useful if we want to investigate the deviation of fixation from the cued directions.

### Analysis of locomotion

To test whether the genetic manipulation in the central complex led to a deficit in locomotion, which in turn contributed to the change in fixation behavior, we analyzed the average movement speed and activity level of the flies. The average speed was calculated by dividing the total movement distance by the total duration of movement in one stage of the task. The activity level was defined as the percentage of video frames in which the fly moved.

### Vision test

We tested the vision of the flies to ensure that the observed behavioral changes were not because of vision impairment. In the first stage of the test, we allowed a fly to freely move on the platform for 60 s. In the second stage, we put a laser spot (50 mW, wavelength = 532 nm) on the platform and gradually moved the spot toward the fly until the laser hit the fly’s body. We repeated this procedure several times. Because of its power, the laser spot was strongly aversive to the flies, and they quickly learned to avoid the laser spot. Typically, after a couple of hits, the flies began to escape the approaching laser spot before it reached them. As a control, we also tested flies with deficient photosensors (genotype: *;:ninaE*; Movie 1) and wild-type flies with amputation of the foreleg (genotype: *w^+^*; Movie 2; Isakov et al., 2016). Such escape behavior would not occur if the flies could not visually perceive the approaching laser spot. We measured the escape rate, *r*, which was defined as:

(11) $$r=\frac{\text{Number of escapes}}{\text{Total number of laser approaching events}}.$$

Movie 1. Laser escaping task for testing the visual perception of the flies with deficient photoreceptors. 10.1523/ENEURO.0537-20.2021.video.1

Movie 2. Laser escaping task for testing the visual perception after amputation of foreleg in the wild-type flies. 10.1523/ENEURO.0537-20.2021.video.2

We then compared *r* between the transgenic flies and the wild-type flies. We would expect a lower *r* for the flies if they were visually impaired.

### Statistical analysis

For wild-type flies, 10–25 individuals were used for each experimental condition in the short-term orientation memory test. For the neural functional experiments and optogenetic activation experiments, 9–20 flies were used in each group.

The statistical analyses were performed using Statistical Product and Service Solutions 22.0 (SPSS 22.0). The *PI* was analyzed using the multi-factor within group analysis of variance, and the differences between groups with different genetic conditions were evaluated using the mixed variance analysis.

### The EB-PB circuit model

We used a previously proposed spiking neural network model of partial central complex (Su et al., 2017), which was built based on the connectomic data of the EB and PB (Lin et al., 2013; Wolff et al., 2015). The model was simulated using the Flysim simulator (Huang et al., 2019). The detailed model equations, neuronal connectivity and model parameters are available in Su et al. (2017). Here, we only provide a concise description of the model. The model network consists of two coupled ring circuits with attractor dynamics (Fig. 1*C*; Extended Data Fig. 1-3*A–F*): (1) the recurrent C circuit (Extended Data Fig. 1-3*G*), formed by EIP (or E-PG) and PEI, is responsible for maintaining a stationary activity bump when a fruit fly moves straight, or when it stops; (2) the shifter P circuit (Extended Data Fig. 1-3*H*), formed by EIP and PEN (or P-EN) neurons (Kim et al., 2017; Turner-Evans et al., 2017; Green et al., 2019), is responsible for shifting/updating the bump in accordance with the body rotation or the motion of a salient visual object so that the bump always indicates the correct head direction with respective to a cued location. Each of the ring circuits is mapped topographically to 360° of the horizontal plane of the external space. The P circuit can be divided into two subcircuits and each corresponds to a unilateral PB. Activation of each subcircuit, or one side of PB, shifts the bump in different directions. The model predicts that alternated activation of the C and P circuits is crucial for a fruit fly because its movement pattern is usually characterized by interleaved straight movement and rotation. The model further predicts that the alternated activation of the C and P circuits is controlled by corresponding GABAergic C-ring and P-ring neurons, which inhibit PEI and PEN neurons, respectively (Extended Data Fig. 1-3*I*). The C-ring neurons regulate the stability of the bump while the P-ring neurons regulate the bump shift. There is an additional type of ring neurons (EIP-ring neurons), which always activate to provide global inhibition that maintains a narrow bump (Extended Data Fig. 1-3*H*). Therefore, to validate the hypotheses of the model, one must experimentally manipulate the bump-position regulating ring neurons and the bump-width controlling ring neurons.

The neurons in the model are simulated using the leaky integrate-and-fire model and the synapses are conductance-based with the exponential dynamics. The interactions between EIP, PEI, and PEN neurons are mediated through excitatory NMDA receptors. Ring neurons inhibit the EIP, PEI, or PEN neurons though GABAergic receptors.

### The behavior model

The main goal of the model was to demonstrate whether the EB-PB circuits are capable of maintaining and updating the activity bump under the given experimental conditions so that an accurate orientation is available to the flies when they want to fixate on the landmarks or the cued directions. The EB-PB circuit model does not control when the flies decide to fixate and how they move their bodies. This steering control is likely to be conducted in the brain regions downstream to the EB-PB circuits (Stone et al., 2017) and are out of the scope of the present study. Therefore, we modelled the behavior of the fly using a simple mathematical model. By analyzing recorded fly behavior in the task, we found that the flies randomly switched between two behavioral states: forward movement and rotation, and the statistical characteristics matched those of the Markov-chain dynamics. Interestingly, we discovered that although the flies performed fixation during the stimulus and poststimulus stages, the overall movement behavior in the two stages still matched the Markov-chain dynamics. This was because the flies in these two stages still randomly switched between the forward movement and rotation states. The fixation was produced by the occasional rotations that were made toward the landmarks. Based on the analyses we implemented a random movement protocol, which consists of two movement states, forward movement and rotation. The model switches between the two states randomly based on the Markov-chain dynamics (see below, The model parameters). In the model, a movement state lasted for a minimum length of one time step (300 ms). At the end of each time step, the fly switches to the other state or remains in the same state based on preset probabilities that are independent of the history. The probability of switching from forward movement to rotation is 0.40 and from rotation to forward movement is 0.60. These numbers were derived by analyzing the distribution of bout length for each movement type in the behavioral tasks (see below, The model parameters).

During the forward movement state, the fly maintains its orientation without turning, while during the rotation state, the fly rotates in a randomly selected direction until the end of the state. The body rotation is accompanied by updating the activity bump in the EB-PB model through the unilateral activation of the PB.

### The simulation protocol

We simulated the second (stimulus) and the third (poststimulus) stages of the behavioral task. In the simulation, the two stages were represented by landmark onset and offset, respectively. In the stimulus stage, the circuit model received visual input from the landmark and the signal about the movement state (forward or rotation) from the behavioral model described above. The visual input was modelled by sending spike input to the PB subregions which triggered an activity bump in the EB that corresponded to the landmark location. For the forward movement state, the P-ring neurons were activated to suppress the P circuit and the C circuit were allowed to operate to stabilize the activity bump in a fixed location. For the rotation state, the C-ring neurons were activated to suppressing the C circuit. At the same time, an input to the unilateral PB was applied to shift the activity bump in the P circuit. Input to the left side of the PB induced clockwise rotation of the activity bump, while input to the right side of the PB induced counterclockwise rotation. This input, presumably originated from the movement feedback, is used to model the observed counter-movement of the activity bump when the fly rotates its body. In the poststimulus stage, the visual input was turned off, while the movement feedback remained. Approximately 200 trials were simulated for each experimental condition.

### The model parameters

The parameters of the EB-PB model mainly follow those used in a previous study (Su et al., 2017), but some parameters were modified in this study (Table 1). The parameters of the behavioral model were determined by the behavioral experiment described in this study. We analyzed the movement patterns of the flies during the first (prestimulus) stage and used them to determine the parameters associated with the forward movement and rotation state. By fitting an exponential curve to the distribution of the forward movement bouts, and the body rotation bouts, we determined the probabilities that a fly switch between the forward movement states and the rotation state. Based on the result, we constructed a Markov-chain model of the random walk behavior of the fruit flies.

**Table 1:** Synaptic weights between neuron types

Source	Destination			
	EIP	PEI	PEN	Ring-EIP
EIP		4.0	4.0	1.0
PEI	8.0/4.0/4.0^*^			
PEN	10.0/5.0/15.0^**^			
Ring-EIP	3.0			1.6
Ring-PEI		10.0		
Ring-PEN			10.0	

* The first and second numbers indicate the synaptic weights of the central connections, e.g., PEI0→EIP10, and the peripheral connections, e.g., PEI0→EIP2. The third number indicates the atypical connections, e.g., PEI7/PEI8→EIP0/EIP17.

** The first and second numbers indicate the synaptic weights of the central connections, e.g., PEN0→EIP10, and the peripheral connections, e.g., PEN0→EIP2. The third number indicates the atypical connections, e.g., PEN7/PEN8→EIP0/EIP17.

There were several parameters to be determined: the activation level of the EIP-ring and P-ring neurons in the ring-neuron suppression and photoactivation experiments. We left these as the free parameters and determined them by matching the resulting bump activity to the observed behavior in experiments. For the simulated flies with suppressed EIP-ring neurons, the input was −0.04 nA, while for the simulated flies with suppressed P-ring neurons, the input was reduced to −0.25 nA. For photoactivation of EIP-ring and P-ring neurons, the inputs were both 0.20 nA.

### Code accessibility

The major parameters are provided in Materials and Methods and Table 1. The full source code and parameter files are available at https://figshare.com/articles/online_resource/eNeuro_2020/13359041.

## Results

### Spatial orientation working memory

To investigate the mechanism of spatial orientation working memory in *D. melanogaster*, we first developed a behavioral task based on Buridan’s paradigm (Götz, 1980; Strauss and Pichler, 1998; Yen et al., 2019; see Materials and Methods; Fig. 1*A*,*B*; Extended Data Fig. 1-2*A*). The task consisted of three stages. In the first (prestimulus) stage, in which no visual landmark was presented, the fruit flies walked randomly without any preferred orientation (Fig. 1*D*; Extended Data Fig. 1-2*B*) and spent most of the time walking along the edge of the arena (Extended Data Fig. 1-2*C*). In the second (stimulus) stage, in which two landmarks appeared at 0° and 180° on the screen, the flies exhibited significant visual fixation behavior by walking back and forth between the two landmarks and spent less time walking along the edge (Fig. 1*E*). This stage tested the ability of flies to orient themselves toward visual landmarks, and no memory was required. In the third (poststimulus) stage, in which the landmarks disappeared, we found that the flies still maintained a behavior preference similar to that in the second stage, although with a weaker tendency of fixation (Fig. 1*F*). The ability of fixation in this stage indicated successful recall of the landmark directions with respect to the fly’s momentary heading, and is therefore an indication of spatial orientation working memory. To determine whether the strength of fixation depends on the stimulus (landmarks) exposure time, we conducted the task using different durations for the stimulus stage and calculated the fixation density *FD*, which indicates the percentage of time the flies spent in fixating on the landmark locations during a stage (see Materials and Methods; Fig. 1*G*; Extended Data Fig. 1-4). We observed that the fixation density in the third stage was larger than that in the first stage for all stimulus exposure times, indicating that fruit flies hold memory about the landmark directions. We can estimate the duration of memory in the third stage by multiplying $PI(=FD_{3rdstage}-FD_{1ststage})$ by the duration of the third stage. This number represents the change of the fixation behavior evoked by the earlier exposure to the stimulus in the second stage, and hence indicates the trace of memory. Taking the condition of a 120-s stimulus stage, for example, *PI* was ∼0.20, the duration of the third stage was 120 s, and the estimated memory duration was ∼24 s. Two interesting properties were discovered in this test: first, *PI*, and hence the estimated memory duration, was positively correlated with the stimulus duration (Fig. 1*G*); and second, the estimated memory duration is surprisingly long considering the stimulus duration was shorter than that used in previous studies (Strauss and Pichler, 1998; Neuser et al., 2008).

### Impairment of spatial orientation working memory by suppressing ring neurons

Next, we investigated how suppressing the EIP-ring or P-ring neurons may affect spatial orientation memory in the behavioral task. We used the same behavioral paradigm as described in the previous section with a 60-s stimulus stage and suppressed the ring neurons by hyperpolarizing the membrane (*;;UAS-Kir2.1*; Hong et al., 2008) or by blocking synaptic transmission (*;;UAS-TNT*; Hong et al., 2008; see Materials and Methods). For EIP-ring neuron (Fig. 2*A*) suppression, we found that both methods led to similar behavioral changes. During the stimulus stage, the flies with suppressed EIP-ring neurons exhibited the same *PI* as did the wild-type flies (Fig. 2*B*). But during the poststimulus stage, *PI* of the flies with suppressed EIP-ring neurons decreased significantly, showing that the flies did not fixate on the landmark locations after their offset (Fig. 2*C*).

10.1523/ENEURO.0537-20.2021.f2-1 Extended Data Figure 2-1 Radar plots of the flies in the control groups (wild type: *w^+^*) and flies with suppressed EIP-ring neurons. ***A***, Wild-type *Drosophila* (genotype: *w^+^*). ***B***, ***C***, Flies with suppressed EIP-ring neurons (***B*** for 32°C, *c105-GAL4, tub-GAL80^ts^;; UAS-Kir2.1* and ***C*** for 32°C, *c105-GAL4, tub-GAL80^ts^;; UAS-TNT*) in the poststimulus stage. Download Figure 2-1, TIF file.

The *PI* plots described above provide information regarding the frequency of movement toward the landmarks versus toward the perpendicular directions. However, it is also important to investigate the overall movement patterns of the flies in all directions. Inspection of the movement trajectories (Fig. 2*D*) and the radar plots (see Materials and Methods) of the wild-type flies and the flies with suppressed EIP-ring neurons (Fig. 2*E*) revealed that the suppression did not produce significant effect in the stimulus stage, indicating that the flies largely maintained their fixation behavior. However, the effect was significant during the poststimulus stage. We discovered that while the wild-type flies exhibited a slight deviation from the actual landmark direction (Fig. 2*F*, top row; Extended Data Fig. 2-1*A*), flies with suppressed EIP-ring neurons exhibited a profound change in the preferred movement direction during the poststimulus stage (Fig. 2*F*, bottom row; Extended Data Fig. 2-1*B*,*C*). This deviation increased with time (Table 2). Flies that underwent two different methods of EIP-ring neuron suppression exhibited similar deviation rates, which were markedly larger than that of the wild-type flies (Fig. 2*G*).

**Table 2:** Fixation deviation and fixation strength of wild-type flies, flies with suppressed EIP-ring neurons, and flies with suppressed P-ring neurons in different time windows of the poststimulus stage

Type of fly	Genotype	Measure of fixation behavior	Time in the poststimulus stage (s)					
			0–15	15–30	30–45	45–60	60–75	75–90
Wild type	*w^+^*	Fixation deviation angle (°)	19.69	30.31	35.21	47.25	51.03	36.47
		Fixation strength	0.24	0.15	0.13	0.16	0.12	0.19
Suppression of EIP-ring neurons	32°C, *c105-GAL4,* *tub-GAL80^ts^;;UAS-Kir2.1*	Fixation deviation angle (°)	27.53	40.64	30.51	58.44	64.89	41.08
		Fixation strength	0.16	0.07	0.12	0.12	0.13	0.13
	32°C, *c105-GAL4,* *tub-GAL80^ts^;;UAS-TNT*	Fixation deviation angle (°)	39.88	41.60	26.66	50.10	60.53	61.57
		Fixation strength	0.11	0.07	0.13	0.11	0.13	0.11
Suppression of P-ring neurons	32°C, *;;VT5404-GAL4,* *tub-GAL80^ts^/ UAS-Kir2.1*	Fixation deviation angle (°)	31.76	44.10	53.72	36.15	48.45	56.40
		Fixation strength	0.09	0.06	0.08	0.05	0.08	0.07
	32°C, *;;VT5404-GAL4,* *tub-GAL80^ts^/ UAS-TNT*	Fixation deviation angle (°)	35.17	46.36	37.77	53.19	51.40	45.52
		Fixation strength	0.10	0.10	0.08	0.10	0.08	0.10

We next investigated the behavioral changes involving suppression of the P-ring neurons using the same UAS lines (*UAS-Kir2.1* and *UAS-TNT*) as used for the EIP-ring neurons (Fig. 3*A*). Whereas both methods of P-ring neuron suppression led to similar behavioral changes, they were distinct from those led by EIP-ring neuron suppression. With suppression of P-ring neurons, the *PI* of the flies decreased significantly in both the stimulus and poststimulus stages (Fig. 3*B*,*C*). By inspecting the movement trajectories and the radar plots, we discovered that the flies with suppressed P-ring neurons moved randomly during the stimulus stage (Fig. 3*D*) and the poststimulus stage (Fig. 3*E*; Extended Data Fig. 3-1).

10.1523/ENEURO.0537-20.2021.f3-1 Extended Data Figure 3-1 Radar plots of flies with suppressed P-ring neurons in the poststimulus stage. ***A***, 32°C, *;;VT5404-GAL4, tub-GAL80^ts^/UAS-Kir2.1*. ***B***, 32°C, *;;VT5404-GAL4, tub-GAL80^ts^/UAS-TNT*. Download Figure 3-1, TIF file.

One may argue that the loss of fixation behavior in flies with suppressed P-ring neurons was not the result of impairment in spatial orientation but was because of deficiencies in other functions such as vision or locomotion. We first tested the vision for the flies with suppressed P-ring neurons by conducting the laser escaping task (see Materials and Methods). We measured the escape rate for the approaching laser beam and found no significant difference between the wild-type flies (71.88%; Movie 3), and the flies with suppressed P-ring neurons (80.95%; Movie 4), while the escape rate of the fruit flies with deficient photosensors (genotype: *;;ninaE*; Movie 1) was 0%, suggesting that P-ring neuron suppression did not impair vision.

Movie 3. Laser escaping task for testing the visual perception of the wild-type flies. 10.1523/ENEURO.0537-20.2021.video.3

Movie 4. Laser escaping task for testing the visual perception of the flies with suppressed P-ring neurons. 10.1523/ENEURO.0537-20.2021.video.4

To investigate whether the motor function was normal in flies with suppressed P-ring neurons, we measured the activity level (percentage of movement bouts in a given period) and speed (mean movement speed in the movement bouts) in the prestimulus stage. We did not observe significant differences between the wild-type flies and the flies with suppressed P-ring neurons (Table 3). The analysis of speed and activity level may not reflect subtle movement impairments such as steering difficulty. Therefore, we performed detailed analysis on the responses of flies in the laser escaping task described above. The flies exhibited three major types of escape maneuvers in the task: detour, turnback, and acceleration. We found that the percentages of each type of escape maneuver are comparable between the wild-type flies and flies with suppressed P-ring neurons (Table 4). For comparison, we also tested flies with steering difficulty by amputating the unilateral foreleg of wild-type flies (Isakov et al., 2016). The flies had tremendous difficulty to escape the approaching laser beam and the escape rate was only 10.37% (Movie 2).

**Table 3:** Activity level and movement speed of wild-type flies, flies with suppressed EIP-ring or P-ring neurons, and flies with photoactivated EIP-ring or P-ring neurons

Wild-type and neural deficits	Genotype					Activity level (%)			Speed (mm/s)		
						Prestimulus stage	Stimulus stage	Poststimulus stage	Prestimulus stage	Stimulus stage	Poststimulus stage
Wild type	*w^+^*					69.78 ± 4.59	69.35 ± 3.32	64.52 ± 3.96	13.18 ± 0.91	12.18 ± 0.70	13.22 ± 0.77
Suppression of EIP-ring neurons	32°C, *c105-GAL4, tub-GAL80^ts^;;UAS-Kir2.1*					81.38 ± 0.57	83.39 ± 1.05	83.91 ± 0.69	7.90 ± 0.25	8.89 ± 0.60	9.01 ± 0.44
	32°C, *c105-GAL4 tub-GAL80^ts^;;UAS-TNT*					82.30 ± 0.78	83.80 ± 0.66	81.49 ± 0.64	8.57 ± 0.41	9.46 ± 0.29	8.43 ± 0.38
Suppression of P-ring eurons	32°C, *;;VT5404-GAL4, tub-GAL80^ts^/ UAS-Kir2.1*					83.33 ± 1.10	84.62 ± 1.44	82.99 ± 1.53	8.44 ± 0.59	9.30 ± 0.84	9.19 ± 0.92
	32°C, *;;VT5404-GAL4, tub-GAL80^ts^/ UAS-TNT*					86.44 ± 1.56	87.51 ± 1.19	85.53 ± 1.04	11.26 ± 1.37	11.91 ± 1.20	10.70 ± 0.80
Neural deficits	Genotype			Activity level (%)				Speed (mm/s)			
				Prestimulus stage	Stimulus stage first 30 s	Stimulus stage last 30 s	Poststimulus stage	Prestimulus stage	Stimulus stage first 30 s	Stimulus stage last 30 s	Poststimulus stage
Photoactivation of EIP-ring neurons	*c105-GAL4;; UAS-CsChrimson.mVenus*			71.57 ± 3.79	66.20 ± 5.39	28.81 ± 4.26	55.84 ± 3.76	12.86 ± 0.81	10.96 ± 0.73	5.80 ± 0.57	9.19 ± 0.80
Photoactivation of P-ring neurons	*;;VT5404-GAL4/UAS-* *CsChrimson.mVenus*			84.65 ± 6.20	85.71 ± 3.79	72.40 ± 7.56	68.42 ± 7.92	15.26 ± 1.01	13.27 ± 0.92	11.88 ± 1.15	13.58 ± 0.82
Neural deficits	Genotype	Activity level (%)					Speed (mm/s)				
		Prestimulus stage	Stimulus stage	Poststimulus stage 0–20 s	Poststimulus stage 20–30 s	Poststimulus stage 30–90 s	Prestimulus stage	Stimulus stage	Poststimulus stage 0–20 s	Poststimulus stage 20–30 s	Poststimulus stage 30–90 s
Photoactivation of EIP-ring neurons	*c105-GAL4;; UAS-* *CsChrimson.mVenus*	68.38 ± 4.19	70.79 ± 4.13	69.87 ± 5.50	34.25 ± 6.84	58.84 ± 4.15	10.41 ± 0.55	10.06 ± 0.65	8.95 ± 0.87	5.98 ± 0.67	8.70 ± 0.57
Photoactivation of P-ring neurons	*;;VT5404-GAL4/UAS-* *CsChrimson.mVenus*	81.32 ± 3.91	77.41 ± 3,42	75.17 ± 4.95	79.71 ± 3.02	76.51 ± 3.07	14.68 ± 0.78	14.29 ± 0.61	13.99 ± 0.87	12.05 ± 0.90	13.27 ± 0.58
Wild type	Genotype	Activity leve (%)	Speed (mm/s)								
Amputating the unilateral foreleg	*w^+^*	71.04 ± 6.66	8.50 ± 0.75								

**Table 4:** The percentages of each type of escape maneuver in the laser escaping task for the wild-type flies and flies with suppressed P-ring neurons

Genotype	Escape behavior	Escape rate (%)
w^+^	Detour	75.00
	Turnback	50.00
	Acceleration	88.89
Suppression of P-ring neurons (*VT5404 > Kir2.1* or *VT5404 > TNT*)	Detour	80.00
	Turnback	50.00
	Acceleration	100.00

### Impairment of spatial orientation working memory by activating ring neurons

We have inspected the effect of the ring neuron suppression, which presumably overactivated the downstream circuits. But how about suppression of these downstream circuits? In particular, if we transiently suppress the circuits, would orientation memory interrupt? To this end, we activated the EIP-ring or P-ring neurons by optogenetics using two temporal protocols (see Materials and Methods; Fig. 4; Extended Data Figs. 4-1, 4-2). For EIP-ring neuron photoactivation during the last 30 s of the stimulus stage, the movement speed and activity level of the flies significantly reduced during this period (Table 3; Movie 5), but the fixation behavior in the poststimulus stage did not exhibit significant difference with that of the wild-type flies (Fig. 4*A*). The result indicated that orientation working memory is not interfered by photoactivation of the EIP-ring neurons during the stimulus stage. When the photoactivation was applied midway during the poststimulus stage (20–30 s), the flies lost the fixation behavior after the photoactivation (Fig. 4*B*). For P-ring neuron photoactivation during the last 30 s of the stimulus stage, the speed and activity level of the flies were close to that of the wild-type flies (Table 3; Movie 6), and the fixation behavior during the activation period remained intact but slightly weakened during the poststimulus stage (Fig. 4*C*). However, photoactivating the P-ring neurons during the 20–30 s of poststimulus stage abolished the subsequent fixation behavior (Fig. 4*D*), an effect that is identical to that of the EIP-ring activation during the same period (Fig. 4*B*,*E*).

10.1523/ENEURO.0537-20.2021.f4-1 Extended Data Figure 4-1 Performance of control groups with the same types of transgenic flies used in the optogenetic experiments. ***A***, ***B***, Performance for flies that carry *c105-GAL4;;UAS-CsChrimson.mVenus*, but without feeding ATR. The schematic protocol (top) and the radar plots for the stimulus stage (bottom left, first 30 s; bottom middle, last 30 s) and the poststimulus stage (bottom right). ***B***, Same as in ***A***, but with photoactivation during 20–30 s of the poststimulus stage. ***C***, Same as in ***A***, but with ATR fed and no photoactivation. ***D–F***, Same as in ***A–C***, but for flies that carry *;;VT5404-GAL4/UAS-CsChrimson.mVenus*. Both control groups exhibited a similar fixation performance to that of the wild-type flies. Download Figure 4-1, TIF file.

10.1523/ENEURO.0537-20.2021.f4-2 Extended Data Figure 4-2 Movement patterns of individual flies in the photoactivation experiments. ***A***, Distribution of the fixation bouts (black bars) of each fly with the photoactivation of the EIP-ring neurons (*c105-GAL4;;UAS-CsChrimson.mVenus*) during the last 30 s of the stimulus stage. ***B***, Same as in ***A***, but with photoactivation during 20–30 s of the poststimulus stage. ***C***, ***D***, Same as in ***A***, ***B***, but for non-ATR fed. ***E***, Same as in ***C***, but for ATR fed and no photoactivation was delivered. Download Figure 4-2, TIF file.

10.1523/ENEURO.0537-20.2021.f4-3 Extended Data Figure 4-3 Same as in Extended Data Figure 4-2, but for flies that carry *;;VT5404-GAL4/UAS-CsChrimson.mVenus*. Download Figure 4-3, TIF file.

Movie 5. A representative trial of EIP-ring neuron photoactivation during the last 30 s of the stimulus stage. The landmarks located outside the movie and were placed at the left and right sides of the arena. 10.1523/ENEURO.0537-20.2021.video.5

Movie 6. A representative trial of P-ring neuron photoactivation during the last 30 s of the stimulus stage. The landmarks located outside the movie and were placed at the left and right sides of the arena. 10.1523/ENEURO.0537-20.2021.video.6

### Hypothesis of the underlying neural mechanisms

To summarize the results of the behavioral and neural functional tests, we plot schematics that illustrate the observed movement patterns of wild-type flies and flies with EIP-ring or P-ring neuron manipulation in all three stages (Extended Data Fig. 5-1). The wild-type flies exhibited strong fixation behavior toward the landmark directions during the stimulus stage. The fixation behavior was still strong but with a slight deviation in the fixation direction during the poststimulus stage, indicating the presence of working memory of the landmark direction (Extended Data Fig. 5-1*A*). Flies with photoactivation of EIP-ring or P-ring neurons during the stimulus stage performed similarly (Extended Data Fig. 5-1*A*). Flies with suppressed EIP-ring neurons also exhibited a strong preference during the stimulus stage (Extended Data Fig. 5-1*B*). However, comparing to the wild-type flies, the deviation increased progressively during the poststimulus stage, suggesting that the flies maintained the memory of the existence of the landmarks but with incorrect memory of their orientation. By contrast, the flies with suppressed P-ring neurons did not exhibit fixation behavior in both stages, indicating that these flies lost spatial orientation regardless the visibility of the landmarks (Extended Data Fig. 5-1*C*). Finally, short photoactivation during the poststimulus stage of either EIP-ring or P-ring neurons both led to abolished fixation behavior after the activation period (Extended Data Fig. 5-1*D*).

**Figure 5.**
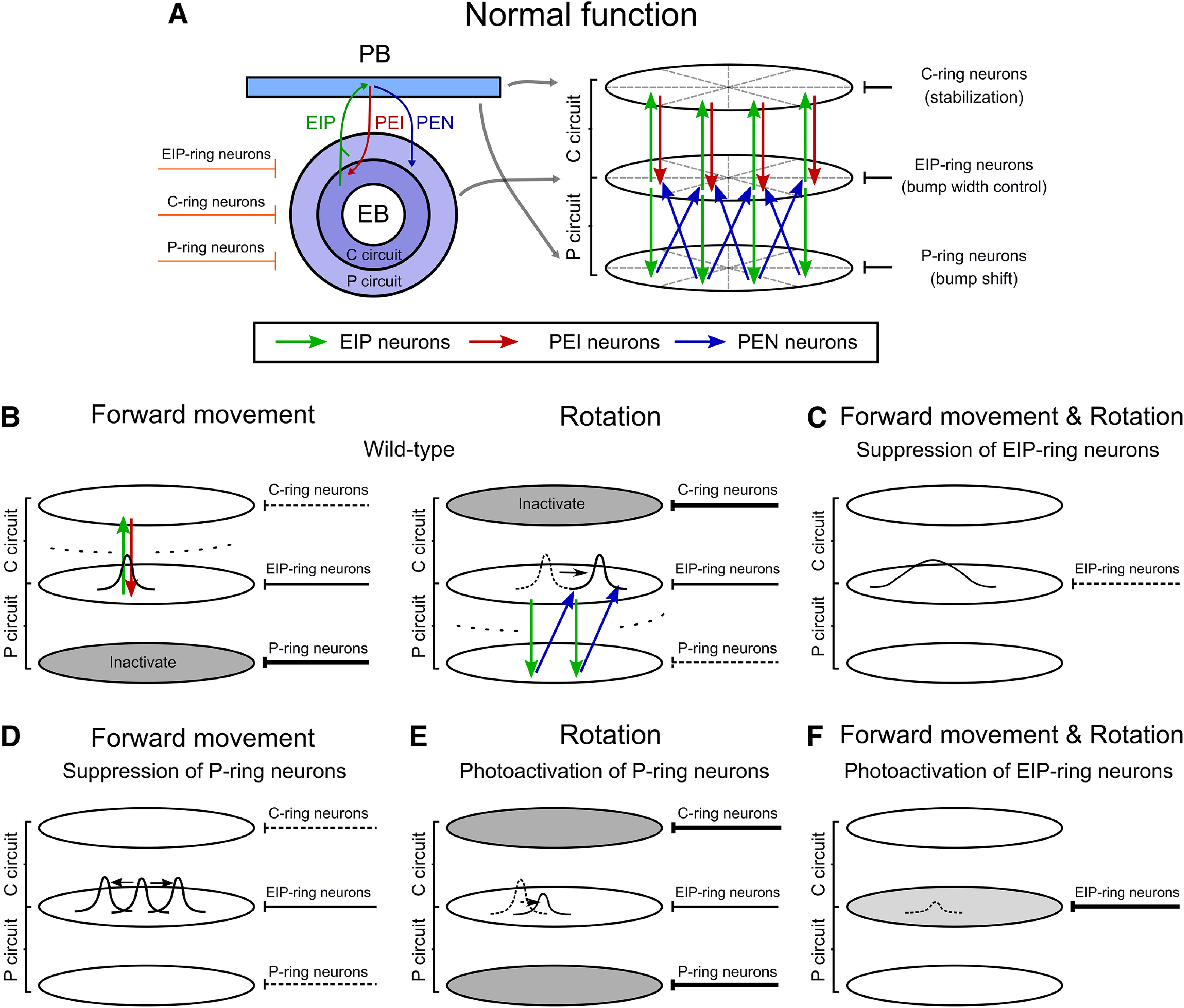
Schematics of the neural activity underlying the behavioral effects of the EIP-ring or P-ring manipulation based on the EB-PB circuit model (Su et al., 2017). ***A***, The EB-PB circuit model consists of a C circuit (EIP and PEI neurons) and a P circuit (EIP and PEN neurons). The former stabilizes the localized neural activity (the bump) that represents the direction of the landmark and the latter updates the memory by shifting the bump when the body rotates. ***B***, When a fly moves forward, the C circuit is activated and the P circuit is inhibited by the GABAergic P-ring neurons (left). On the other hand, when the fly rotates, the C circuit is inhibited by the GABAergic C-ring neurons and the P circuit is partially activated (right). The GABAergic EIP-ring neurons are always activated to maintain the width of the bump through the global inhibition. ***C***, When the EIP-ring neurons are suppressed, the activity bump becomes wider and drifts more, which leads to an inaccurate orientation memory. ***D***, When the P-ring neurons are suppressed, the P circuit becomes fully activated even during forward movement. The overactivation leads to a destabilized bump that diffuses throughout the ring and the fly loses its orientation. ***E***, The photoactivation of the P-ring neurons suppresses the P circuit, which loses the activity bump during rotation. ***F***, The photoactivation of EIP-ring neurons produces overly powerful global inhibition that suppresses the activity bump during the poststimulus stage when no landmark input is available to support the bump. Schematics of the observed behavior changes is shown in Extended Data Figure 5-1.

The behavioral results can be explained by the neural mechanisms proposed in the EB-PB model (Su et al., 2017; Fig. 5; see Materials and Methods for a brief description of the model mechanisms). To visualize the effects of neural manipulations on the circuits, we simplify the model diagram into an abstract three-ring representation and each ring is modulated by one of the three ring neuron types (C-ring, P-ring, and EIP-ring; Fig. 5*A*,*B*).

10.1523/ENEURO.0537-20.2021.f5-1 Extended Data Figure 5-1 Schematic that summarizes the behavioral effects of EIP-ring and P-ring neuron suppression and photoactivation. ***A***, Wild-type flies maintain fixation behavior in both the stimulus stage and the poststimulus stage, in which the flies exhibited a slight deviation in the fixation direction. Flies with transient photoactivation of the EIP-ring or P-ring neurons during the stimulus stage exhibited a similar behavioral pattern. ***B***, Flies with suppressed EIP-ring neurons exhibited fixation behavior with large deviation in the poststimulus stages, respectively. ***C***, Flies with suppressed P-ring neurons could not maintain fixation behavior, indicating loss of spatial orientation. ***D***, Flies with transient photoactivation of the EIP-ring or P-ring neurons during the early poststimulus stage eliminated the fixation behavior. Download Figure 5-1, TIF file.

Based on the model, if the EIP-ring neurons are suppressed, the reduction of inhibition leads to a broadened bump with jittered position (Fig. 5*C*), which makes the orientation memory less accurate (Extended Data Fig. 5-1*B*). When the P-ring neurons are suppressed, both clockwise and counterclockwise part of the P circuit becomes activated, shifting the bump toward both directions. In consequence, the neural activity spreads through the entire ring (Fig. 5*D*), and the fly loses its orientation completely (Extended Data Fig. 5-1*C*). If we photoactivate P-ring neurons during the poststimulus stage, then the P circuit is suppressed even during body rotation when the C circuit is also inhibited. Therefore, the bump cannot be sustained because of the lack of recurrent excitation from the C and P circuit (Fig. 5*E*). As a consequence, the fly loses its orientation memory (Extended Data Fig. 5-1*D*). Finally, if the photoactivation is performed on the EIP-ring neurons during the poststimulus stage, it would diminish the activity bump because of the excessive inhibition (Fig. 5*F*). Therefore, the fly loses its orientation memory in the third stage (Extended Data Fig. 5-1*D*). However, if the photoactivation is performed during the second stage in which the landmarks are visible, the bump could still be maintained by the input corresponding to the visual cue (Extended Data Fig. 5-1*A*).

### The computational model of spatial orientation working memory

We have illustrated in Figure 5 how interrupted functionality of the C or P circuits affected the spatial orientation working memory based on our basic understanding of the dynamics of the EB-PB model (Su et al., 2017). Next, we further demonstrated such neural mechanisms by performing the model simulations based on the same experimental protocols used in the present study. The model consists of the EB-PB neural circuit model and a Markov-chain behavioral model (see Materials and Methods; Extended Data Fig. 6-1).

**Figure 6.**
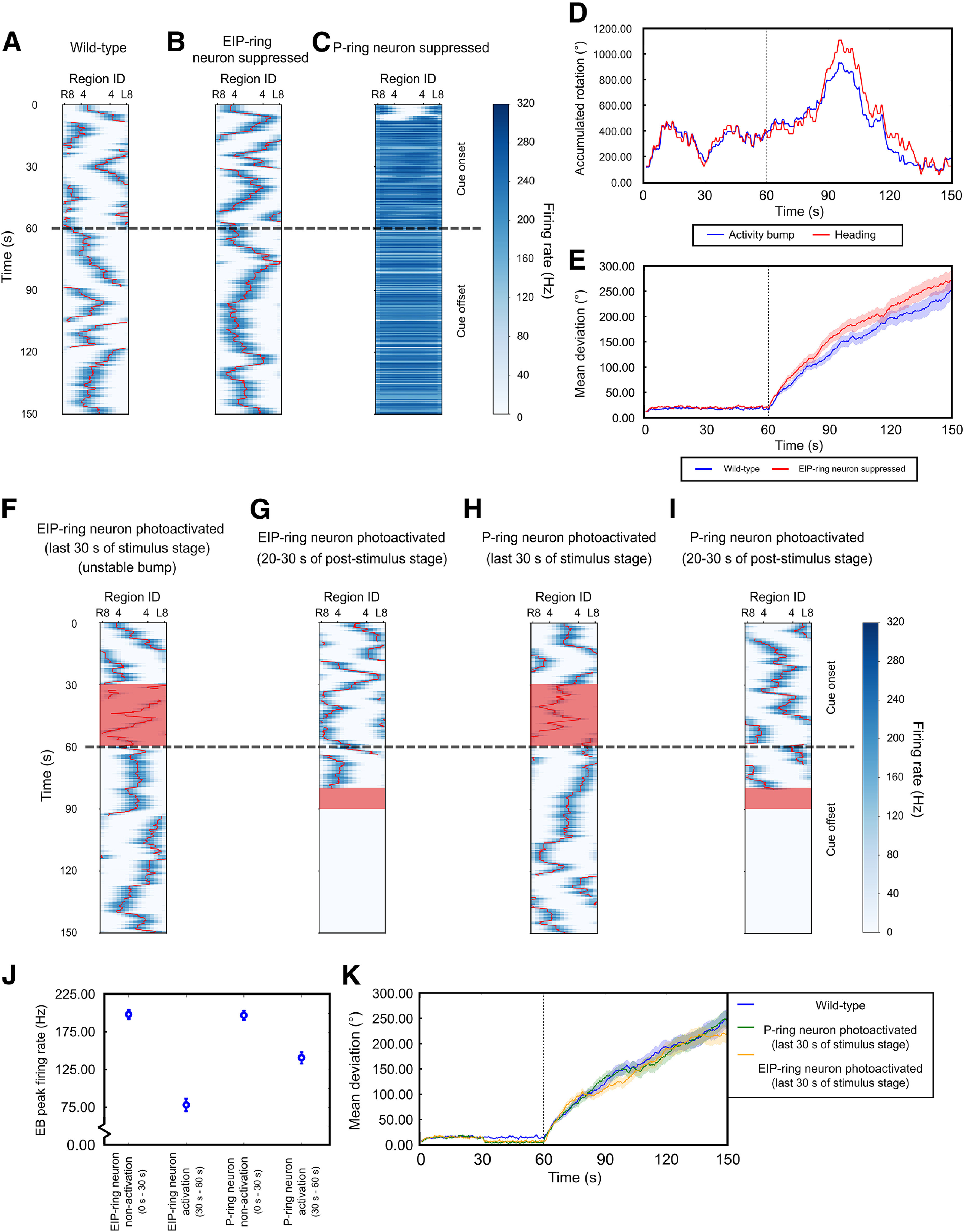
The computer simulations of the EB-PB model (Su et al., 2017) demonstrated the neural mechanisms illustrated in Figure 5, and highlighted the roles of the EIP-ring and P-ring neurons in the spatial orientation working memory. ***A***, The neural activity of EIP neurons in all EB regions as a function of time indicates the existence of activity bump (dark blue) in a simulated wild-type fly. The landmark was on between *t* = 0 s and *t* = 60 s (dashed line). ***B***, ***C***, Same as in ***A***, but with simulated EIP-ring (***B***) and P-ring neurons (***C***) suppression. The P-ring neuron suppression led to unstable and widespread neural activity. ***D***, The accumulated angles of the true heading and the activity bump of in one example trial for a simulated wild-type fly. ***E***, The trial-averaged angular deviation between the head direction and the bump for simulated wild-type flies (blue) and simulated flies with suppressed EIP-ring neurons (red). Shades represent the SEM. ***F***, ***G***, Same as in ***A***, but with simulated photoactivation (red regions) of the EIP-ring neurons. After the photoactivation in the last 30 s of the stimulus stage (***F***), the activity bump persisted in all trials. The photoactivation in the poststimulus stage abolished the activity bump (***G***). ***H***, ***I***, Same as in ***F***, ***G***, but with the photoactivation of the P-ring neurons in the last 30 s of the stimulus stage (***H***) or during the poststimulus stage (***I***). ***J***, EB peak firing rate with no-photoactivation and photoactivation of EIP-ring or P-ring neurons in the stimulus stage. ***K***, The trial-averaged angular deviation between the heading and the bump for simulated wild-type flies (blue), P-ring neuron photoactivation (green), and EIP-ring photoactivation (red). Shades represent the SEM. The behavior model used to drive the EP-PB circuit model is shown in Extended Data Figure 6-1.

We first showed that the model with default settings (corresponding to wild-type flies) was able to maintain activity bump in both stimulus and poststimulus stages as expected (Fig. 6*A*). The model with suppressed EIP-ring neurons still exhibited a clear activity bump (Fig. 6*B*) in both stimulus and poststimulus stages while P-ring neuron suppression lost the activity bump completely in both stages (Fig. 6*C*). However, compared with the wild type, although the bump was maintained in the condition of EIP-ring neuron suppression, the bump width [full-width at half-maximal (FWHM) = 2.04 ± 0.04 rad] is larger than that of the simulated wide-type flies (FWHM = 1.86 ± 0.05 rad). Similar to the real flies, the simulated wild-type files exhibited progressively increasing deviation between the bump position and the head direction during the poststimulus stage (Fig. 6*D*). Moreover, the bump with EIP-ring neuron suppression drifted more during the poststimulus stage and the mean deviation between the true heading and the activity bump location was larger than the simulated wild-type fly (Fig. 6*E*). The simulated result is consistent with what are pictured in Figure 5*B–D*, and explained the observations shown in Figures 2, 3. Flies with suppressed EIP-ring neurons still performed robust fixation activity because of the existence of the activity bump but with less accurate fixation direction because of the larger deviation between the true heading and the bump position. On the other hand, flies with suppressed P-ring neurons did not fixate because of the loss of the activity bump.

10.1523/ENEURO.0537-20.2021.f6-1 Extended Data Figure 6-1 Distributions of duration of different behavioral states and the Markov-chain model of behavioral control. Dashed lines represent the fitting curves, which is given by $y=\alpha *\beta^{\frac{x}{0.3}}$. α is the fitting parameter and represents the probability of staying in the same state in each time window of 0.3 s. The exponential shape of the distribution of state duration indicates the Markov-chain dynamics. ***A***, The distribution of duration of the rotation state in prestimulus stage. α = 0.473, β = 0.533, and $R^{2}$ = 0.986. ***B***, Same as in ***A*** but for the forward movement state, α = 0.657, β = 0.448, and $R^{2}$ = 0.985. ***C***, The distribution of duration of the rotation state in stimulus stage. α = 0.482, β = 0.529, and $R^{2}$ = 0.978. ***D***, Same as in ***C*** but for the forward movement state, α = 0.688, β = 0.439, and $R^{2}$ = 0.984. ***E***, The distribution of duration of the rotation state in poststimulus stage. α = 0.495, β = 0.523, and $R^{2}$ = 0.975. ***F***, Same as in ***E*** but for the forward movement state, α = 0.621, β = 0.464, and $R^{2}$ = 0.984. ***G***, The behavioral model was constructed based on the Markov-chain dynamics and described the probability (given by the numbers next to the arrows) of a fly switching between behavioral states. Download Figure 6-1, TIF file.

Next, we simulated the photoactivation experiments in the model. The EIP-ring neuron activation during the last 30 s of the stimulus did not affect the activity bump during the poststimulus stage (Fig. 6*F*), while photoactivation during the poststimulus stage completely abolished the bump (Fig. 6*G*). This is consistent with the picture depicted in Figure 5*F* and the experimental observation (Fig. 4*B*). Interestingly, P-ring neuron activation in the simulations produced similar effects (Fig. 6*H*,*I*) but with two subtle differences. First, when EIP-ring or P-ring neurons were activated during the last 30 s of the stimulus stage, the strength of the bump, as measured by the peak firing rate of the bump, was both reduced. But the reduction is much more significant for the activation of EIP-ring neurons than for the P-ring neurons (Fig. 6*J*). We do not know how this difference may be reflected at the behavioral level, but we did observe that the activity level during the period of photoactivation was lower in the EIP-ring neuron activation condition than in the P-ring neuron activation condition (Table 2, bottom two rows) in experiments. However, we stress that the causal relationship between the bump strength and the activity level is just a speculation and is not a prediction of the EB-PB model. Second, with P-ring neuron photoactivation during the second stage, we found that the bump could be lost in a small percentage (24.2%) of trials. This is because of the instability occurring when the P circuit recovered from the inhibition at the end of the photoactivation. The instability might result from the model implementation or the choice of model parameters, and might not reflect an actual neuronal phenomenon. Finally, for either EIP-ring or P-ring neuron activation, if the bump was maintained during the poststimulus stage, the deviation between the true heading and the bump location is comparable to that in the simulated wild-type flies (Fig. 6*K*). This is consistent with the observation that the flies in these two conditions performed as good as the wild-type flies during the poststimulus stage (Fig. 4*E*).

## Discussion

In the present study, we hypothesized that the C circuit and P circuit in the EB circuits stabilize and update the orientation-encoding activity bump and they are regulated by corresponding GABAergic ring neurons. We tested our hypothesis by manipulating two types of GABAergic ring neurons in a spatial orientation working memory task with free moving fruit flies, and discovered manipulating each ring neuron type led to different behavioral abnormality. By performing computer simulations on a previously proposed EB-PB neural circuit model, we were able to explain the results of the experiments and provided a picture of the neural circuit mechanism underlying spatial orientation working memory: the orientation-encoding bump is maintained through two alternately activated neural processes: one that stabilizes the position of the activity bump and one that updates the position of the bump. The former is activated when a fruit fly maintains a steady head direction and the latter is activated when the fly rotates its body. The control of this process is performed through specific GABAergic ring neurons. Therefore, overactivating or suppressing the ring neurons disrupts the alternation of the two processes and leads to incorrect or even loss of orientation memory.

There are a few more interesting discoveries worth discussing. Performing fixation toward previous landmark directions requires two things to be remembered: the earlier event of the landmark presentation (what) and the directions of the landmarks (where). Flies that fail to remember the former would not exhibit the fixation behavior at all, while flies that forget the latter would still perform the fixation but toward incorrect directions. Our discoveries of strong fixation but with large deviation from the true directions of the landmarks for flies with EIP-ring neuron suppression during the third stage (Fig. 2*F*,*G*) may imply the segregation of the neural mechanisms of orientation memory regarding the “where” and “what” of a landmark.

One interesting finding of the present study is a long duration of spatial orientation working memory during the poststimulus stage. Previous studies reported the occurrence of poststimulus fixation behavior that lasted only for a few seconds immediately following the offset of the landmarks (Neuser et al., 2008). Indeed, we observed that the flies tended to stop their movement a few seconds after the sudden disappearance of the landmarks in the third stage. But they usually resumed the movement in a few seconds. This might be the reason why earlier studies only claimed a few seconds of fixation if their analyses did not include the resumed movement. Further studies are needed to investigate this issue.

A couple issues regarding the choice of molecular tools should be discussed. In the present study we used *tub-GAL80^ts^* in combination with *UAS-Kir2.1* or *UAS-TNT* to suppress targeted ring neurons. The method involves raising the temperature 1 d before the behavioral experiments and therefore taking effects on a much longer time scale than using optogenetic tools. This long-term suppression may induce other effects at the cellular or circuit levels, which are beyond what our model can simulate. Further study may be required to carefully examine the long-term effects. An ideal solution is to transiently suppress the ring neurons using optogenetic tools such as *UAS-NpHR* (peak sensitivity wavelength ≈589 nm; Deisseroth, 2010) or *GtACRs* (peak sensitivity wavelength ≈ 527 nm for *GtACR1* and ≈457 nm for *GtACR2*; Mauss et al., 2017). However, the wavelength of the required activation light is within the visible range of the fruit flies. Our preliminary tests on *UAS-NpHR* showed that the onset of the activation light seriously disrupted the fixation pattern of wild-type flies. A new optogenetic tool or a carefully re-designed optical system is required to transiently suppress targeted ring neurons while not interfering the visual experiments. The second issue is related to the *GAL4* lines. In the present study we only used two most specific lines, *c105-GAL4* and *VT5404-GAL4*, to target the EIP-ring and P-ring neurons, respectively. As listed in the Methods section, there are several other less specific *GAL4* lines available for the two types of neurons. It is necessary to conduct the same experiments using these overlapping lines to further confirm the results presented in this study.

Several other important questions remain to be addressed. Previous studies showed that EB does not maintain a fixed retinotopic map and a bump can start from a random location in the beginning of a trial (Seelig and Jayaraman, 2015). For the sake of modeling simplicity, we did not model the random starting point feature in our model. But this feature is easy to implement and does not affect the conclusion of this study. We simply need to apply a global excitation to the entire EB to reset the system. The excitation will induce strong competition between the EIP neurons and a new bump will start at a random location through the winner-take-all dynamics. Following this issue, random regeneration of an activity bump also needs to be discussed. Our model showed that photoactivation of either EIP-ring or P-ring neurons during the poststimulus stage permanently abolished the activity bump. However, based on the observation of spontaneous generation of activity bump in other studies (Seelig and Jayaraman, 2015), the bump is likely to be regenerated at a random location after the offset of photoactivation. The regeneration can be easily implemented in our model using the same mechanism described above. Since the regenerated bump starts from a random location, the fruit flies lose the reference to the landmark locations. Thus, adding a spontaneous bump or not both lead to the same conclusion: the files fail to fixate on the previous landmark locations. Although not affecting the conclusion of the present study, the spontaneous bump feature may be crucial in future studies that involve modeling of the steering mechanism.

Another issue is that a couple experimental and modeling studies suggested that ring neurons provides the mechanisms underlying flexible retinotopic mapping in EPG (or EIP) neurons rather than the simple suppression/activation mechanism as hypothesized in the present study (Fisher et al., 2019; Kim et al., 2019). However, the ring neurons (R2 and R4d) tested in one study (Fisher et al., 2019) are of different types from what we tested (R1 and R6) here. Additional experiments that measure the activities of R2 and R4d using the setups described in Fisher et al. (2019) or Kim et al. (2019) are required to clarify this issue.

It is important to compare and discuss differences between computational models of the central complex in terms of the functions investigated in the present study. However, most models focused on different aspects of the compass circuit functions (see Introduction). Among these models the one proposed in Kakaria and de Bivort (2017; and was also used in Pisokas et al., 2020) can be compared directly to one used in Su et al., 2017 and the present study. Both models used biologically realistic spiking neurons and the underlying circuit mechanisms are comparable. The major difference is that Kakaria and de Bivort model proposes the PB intrinsic neurons as the main source of inhibition that regulates the attractor dynamics, while in our model this function is conducted by the EIP ring neurons with two additional ring neuron types (C-ring and P-ring neurons) modulating different subcircuits of the system. An in-depth model comparison and experimental manipulation of PB intrinsic neurons and ring neurons under the present behavioral task may be able to clarify this issue.

A final issue is related to the function of the activity bump which is commonly thought to represent the fly’s sense of orientation in a manner similar to that of the head-direction system found in rodents (Muller et al., 1996). However, as aforementioned “what” and “where” mechanisms, performing the fixation behavior as an indication of orientation working memory may require several serial or parallel neural components beyond EB and PB. For example, how is this innate fixation behavior initiated (motivation)? When a fly stops fixating, it is not clear whether the fly forgets the landmark directions or simply enters a different behavior state (but still remembers the landmark directions). It also remains unclear whether the memory is stored in another neural circuit and the EB merely provides a reference frame for orientation, or whether the activity bump in the EB represents the actual memory of the landmarks. The current experimental setup is not able to address this issue. A novel task that can disassociate these two components is required for further investigation.

We conclude the present study as follows. First, the experiment indicated that long-term suppression of EIP-ring neurons reduced the accuracy of orientation working memory (fixation with an increased deviation angle), whereas long-term suppression of P-ring neurons abolished the memory completely (no fixation). Similarly, transiently activating either ring neuron types in the absence of landmark immediately abolished the memory. Second, the experimental observation can be explained by the EB-PB neural circuit model in which the EIP-ring neurons are responsible for controlling the width of the bump and the P-ring neurons are responsible for shifting (updating) the position of the bump. Third, put the experiment and the theory together, the present study suggests that coordinated activation of the two ring neuron types which control the downstream EB-PB subcircuits is crucial for spatial orientation working memory.

## Synthesis

Reviewing Editor: Niraj Desai, NINDS Decisions are customarily a result of the Reviewing Editor and the peer reviewers coming together and discussing their recommendations until a consensus is reached. When revisions are invited, a fact-based synthesis statement explaining their decision and outlining what is needed to prepare a revision will be listed below. The following reviewer(s) agreed to reveal their identity: Barbara Webb.

The reviewers and I were impressed by this study and believe it makes a valuable contribution.

Even so, as you can see in the reviews (appended below), both reviewers had some concerns about how the model is presented and used. It is important that readers, even those not intimately familiar with Su et al. (2017), be able to follow and judge your arguments. It is also important to contrast the model with other possible interpretations. Both reviewers offered thorough and careful comments on these and other points.

REVIEW #1

The authors present a follow-up to their 2017 study, where they modelled ellipsoid body (EB) and protocerebral bridge (PB) circuits in Drosophila, which have been shown to be involved in navigation and spatial orientation. In that study (which involved modelling spiking neurons in circuits deduced from recent connectome work), the same group proposed coupled symmetric and asymmetric circuits controlling orientation memory to a visual stimulus and feedback correction following body rotation (in the dark). That purely theoretical work made key predictions about how activity within these feedback circuits might govern spatial orientation. In the present work, the authors test some of these predictions by inhibiting or activating upstream inhibitory ring neurons. Crucially, they test this in freely walking flies, something that hasn’t been done before (all previous work was in tethered animals, complicating any assessment about self-motion and the contribution of ‘P-ring’ neurons towards correcting the position of the visual response in in the EB. The authors employ a version of Buridan’s paradigm, where flies walk back and forth between two visual stimuli, which they test in three phases: no stimulus, stimuli, and visual memory. Achieving consistent behavioral results for their inhibition/activation experiments (on two sets of neurons, c105-Gal4 or EIP-ring and VT5404-Gal4 or P-ring), they return to their 2017 model to observe their predictions are borne out. They are, quite admirably so. I have little to comment on the modelling work in the latter part of the manuscript, this seems to follow logically from their previous work, and the conclusions make sense.

This is a valuable contribution to this growing area of research, although a somewhat difficult paper to read. This is primarily because the average reader will not have read Su et al 2017 (I had to go back and read it carefully to understand this work), so without that background it is quite hard to follow. I have some suggestions on how the study could be made more accessible to the average reader, as well as some questions about the methods and results. These should be addressed before considering publication in eNeuro.

Major suggestions

1. Although references to Su et al 2017 are made, it would really help the paper if some schematic circuit model was presented up front, so that readers could appreciate where the manipulated EIP and P ring neurons reside in the EB/PB context. Why were these the most interesting neurons to manipulate in this study? Also, some better validation may be needed for why c105-Gal4 and VT5404 are the best drivers for these neurons (why were other Gal4s not tested to confirm, especially for VT5404 which seems more non-specific?).

2. Any locomotion towards a stimulus after it is turned off is viewed as visual memory. Surely, flies have some walking inertia and will keep on walking straight irrespective of whether a visual was there or not. The authors support their view that this is memory by finding a significant correlation between prior stimulus duration and ongoing fixation after the stimulus disappears. They demonstrate 40s fixation duration for a stimulus that was on only 30s, and 80s fixation for a stimulus that was on for 2 minutes. That seems long, and I question their fixation metrics (the example in Figure 1 D/E certainly do not look like the tracks of a fixating fly. This may have to do with the ∼17% threshold for fixation ‘anywhere’ as opposed to the actual two objects? This needs better explanation. Also it is unclear why the pre-stimulus ‘fixation durations’ increase with stimulus duration, in Figure 1F. There is no stimulus in the pre-stage, so this makes little sense and this first result may confuse the average reader.

3. There are multiple different fixation readouts: PI, percentage, and the ‘radar plots’. The radar plots seem most informative as they indicate angle deviation from the targets. However, the way they are shown it appears as if their polarity matters (left vs right of the targets), but it probably does not, it’s just an angle deviation. This may also confuse readers. Additionally, conclusions are made using these radar plots when it is unclear if significance was tested (e.g., line 395). Together, it is unclear why there are many different ways of assessing fixation.

4. In Figure 4, a ‘percentage’ version of fixation is employed. This seems to be continuous measure based on the ∼17% threshold based on 6 (?) sectors (anything above 17% being non-random). This metric is hard to appreciate, as it is not clear how a fly can be determined to be fixating or not fixating from one moment to the next just by this threshold. Fixation seems to require time to reveal itself as such, and isn’t an instantaneous readout (at least for a fly in this kind of assay). Extended Although Figure 1-2 provides a level of explanation for how these ongoing fixation measures are determined, this metric remains problematic because the conclusions from Figure 4 simply sum this as a binary readout to propose that activation of both circuits during the memory phase abolishes fixation. From the data in Figure 4 D&H it does not look like fixation was abolished. It is hard to reconcile the conclusions with what can be seen on the graphs, and this may have to do with lack of clarity on what is meant by fixation duration and how it is calculated.

5. Chrimson activation during the third experimental stage abolishes fixation, but activation during the stimulus does nothing. Could this be simply because in the absence of the visual stimulus the red light is distracting, rather than it having an actual circuit-level effect? Was control experiment performed for non-ATR fed flies to control for the effect of red light, with and without the visual stimulus?

6. Although the authors made a good effort at using conditional approaches for suppression (Gal80), these remain chronic manipulations compared to optogenetics, so it is possible that long-term changes in circuit activity do not quite reflect what is being dynamically modelled. This could be mentioned in the discussion.

7. The authors admit in the discussion that the ‘bump’ in the EB isn’t retinotopic, while their model requires a level of retinotopy. How a ‘random’ starting point for the bump might be implemented in their model could be discussed, as this seems a weakness in the model that does not reflect what has been observed.

Minor points and corrections

Language in the abstract needs work, e.g. the 3rd sentence.

Line 199: switches

Line 361: was

Line 430: suppression ‘of’

Line 442: close

Line 446: Figure 4J does not seem to me called out.

Line 498: ‘that how’

Line 509: Should 6B be 6C?

Line 513: Should 6C be 6D?

Line 514: Delete 6D?

Line 580: Please explain what you mean by the ‘brief interruption’ of fixation and a ‘startle’ effect. This is not evident from the figures.

Line 590: When a fly stops fixating...

Line 591: Perhaps provide a reference or two for when you suggest a fly loses motivation or attention. Do flies even have attention?

Figure 1F: There is room on the figure to clarify what you mean by ‘scaled’. This seems important because it is not at all clear why a fly would be fixating if there are no visuals.

Extended Data 2-1: Please explain why there is loss of fixation at 15-30s specifically, in B&C.

REVIEW #2

This paper reports some well-executed experiments to block or activate specific neurons in the CX of Drosophila during a free-walking short-term memory task. The results are of general interest but the interpretation seems overly focused on one specific model, without appropriate consideration of alternatives. This wider context would need to be addressed to make the paper acceptable.

Major issues:

The paper focusses on the ellipsoid body activity bump as a phenomenon that tracks the heading of the animal relative to the world, but neglects the likely function of this bump, i.e., to control moment by moment steering towards a current or remembered goal. Existing models [e.g. Stone et al. 2017] have indeed demonstrated that the circuitry is consistent with a steering mechanism, so it seems very strange that this paper models the actual behavior of the animal as a Markov chain of straight walks and random rotations.

This carries over into the interpretation of the compass circuit itself, in the assumption that there must be separate, alternatively activated circuits for holding the bump steady (for forward walking) and for moving the bump (during rotation). The ring neurons investigated are then assumed to be acting as gating the activity of one or other of these ‘contradictory’ subcircuits. But again, existing models [e.g. Kakaria et al. 2017, Pisokas et al. 2020] show that it is possible for the compass circuit to encompass both properties without gating, that is, to have sufficient recurrent feedback for bump stability, including in the absence of input, but sufficient sensitivity to input to alter the bump position, by appropriate parameter tuning.

The interpretation of ring neuron function also seems somewhat inconsistent with current evidence (although it is true the function is not fully understood). Specifically, the evidence [e.g. Kim et al. 2019, Fisher et al. 2020] would seem to suggest that ring neurons carry information about directional sensory cues (from vision, wind and possibly other modalities) and thus contribute to the location of the bump, possibly through adaptable synaptic connections, rather than just acting as general inhibition.

In summary, there remains enough uncertainty about this circuit that the authors are welcome to propose their own interpretation, and argue for support in their data, but this needs to be defended in relation to alternative models and potentially contradictory evidence, not presented as though it is the only solution.

In addition, the actual presentation of the computational model is inadequate. How many units are simulated in the model? Using what kind of neural model? What is the exact connectivity it encapsulates? How do the ring neurons interface? If I understand correctly, the model does not actually model the behavior of the animal, such as the steering observed when visual stimuli are present. Yet the closed loop interaction of the circuit with behavioral control is key to understanding function. Indeed this is also quite critical to bridge their interpretation of the (possible) effects of the manipulations on the bump characteristics to the observed behavior of the animal.

As such, the final conclusions of the paper are not well supported. For example, it could be argued that all the effects demonstrated relate to the extent to which a stable bump can be maintained in the absence of external orienting stimuli, rather than to “crucial roles of two types of GABAergic ring neurons in spatial orientation working memory”. Their model does not include mechanisms of either memory or behavior, but only shows phenomenological effects on the bump following the removal of stimuli; not “a detailed picture of how the global inhibition (from the ring neurons) modulates the memory”.

Minor issues:

There are a number of odd citations (possibly something has become confused?) E.g. line 34, these seem an odd choice of citations for ‘neuromorphic engineering’ and at line 36, Dewar et al. seems inappropriate.

Lines 45-47, there are additional models of the CX that should be cited.

Lines 55-56, the underlying theory of attractor circuits (which should be cited) has long been able to account for both stability and updating of the bump - there is no need to conceive of them as contradictory mechanisms.

Line 65-70 It is unclear how the prediction of the Su et al model was in any way specific or how it was verified by the study of Turner-Evans et al.

Line 212 it seems misleading to call this the ‘simulated behavioral task’ when the control of behavior is not simulated. I became quite confused at this point in the manuscript, assuming the simulated fly must be somehow directed towards the stimulus or the bump memory, and only understanding on line 500 in the results that this is not the case.

Line 266-267, the definition of fixation duration seems extremely permissive, that is, counted as any time (in a 15s sliding window) the fly movement direction is between [-30,30] or [150,210] degrees more than the pure chance level of 1/6 of the time. As such it is maybe not so surprising that they see ‘unexpectedly long” duration of the behavior? From Fig 1, it appears flies spend a substantial amount of their time (around 1/3) ‘fixating’ during the pre-stimulus stage, by this measure. It is not clear in the later discussion (line 523 on) if the measure used here is actually comparable to that used in previous studies.

Line 375 “While the flies with suppressed EIP-ring neurons exhibited the same PI as did the wild-type flies during the stimulus stage (Fig. 2B), the PI of the flies with suppressed EIP-ring neurons decreased significantly during the post-stimulus stage and was markedly lower than that of the wild-type flies (Fig. 2C).” How is this possible when PI is defined (line 269) as the difference between the third and first stage? I assume the PI can also be difference between second and third stage, but this needs to be made clear in the methods.

Line 478 on, “the EP-PB model is only a neural circuit model and does not include any behavioral component. Nevertheless, we can still infer the behavioral outcomes...” This speculation would be much more convincing if a behavioral component was included in the model, to demonstrate that the changes in bump activity (e.g. broader width, greater drift) actually do result in the behavioral effects that were observed.

Line 521 on, it seems surprising in the model results that the ‘photoactivation’ causes the bump to be permanently abolished (see also fig 6), i.e., without recovery after the additional inhibition is removed . In most ring attractor models, a bump will form spontaneously (in the absence of input that influences the bump position).

Line 535 “when the P-ring neurons were activated in the last 30 s of the stimulus stage, the activity bump was lost in the subsequent post-stimulus stage in a small percentage (29 out of 120) of the trials”. What factor in the simulation causes this non-deterministic result? How was the proportion affected by the tuning of the parameter representing photoactivation (line 246)?

The grammar in the paper also needs some improvement, here are some of the errors noted:

Lines 5-6, this is ungrammatical: “However, how does this system involved in the orientation working memory at the behavioral level is not well understood.” -> “How this system is involved”.

Line 73 Problem with tense, it should be “in particular those involved in stabilizing and updating”.

Line 142 Problem with tense, it should be “to transiently activate EIP-ring”.

Line 361 grammar: “the third stage were longer than the that in the first stage", maybe change this to “the third stage was longer than that in the first stage”.

Line 430 grammar, maybe change it something like this “suppression of these downstream”.

Line 438 grammar: the word “interference” is not used properly.

Line 442 spelling should be “the flies were close to”.

Line 498: remove “that”.

Line 598 “in three aspects”. The following text lists four aspects.

## Author Response

Dear Niraj Desai,

We thank the editor and reviewers for the constructive comments that greatly help us to improve the manuscript. We have made substantial changes to the manuscript to address all the concerns. The revision includes modifying four figures, six extended data figures and added/modified ∼320 lines of text (highlighted). We also provide point-by-point responses as follows.

Synthesis Statement for Author (Required):

The reviewers and I were impressed by this study and believe it makes a valuable contribution.

Even so, as you can see in the reviews (appended below), both reviewers had some concerns about how the model is presented and used. It is important that readers, even those not intimately familiar with Su et al., (2017), be able to follow and judge your arguments. It is also important to contrast the model with other possible interpretations. Both reviewers offered thorough and careful comments on these and other points.

Reply: We have added a new figure panel (Figure 1C) to illustrate the basic idea of the Su et al., 2017 model, and replaced Figure 5 to show more detailed model mechanisms and also to show how the model explains the deficits associated with different neural manipulation performed in the study. We have also rewritten part of the Methods (lines 285-309) and Results (lines 512-532) to explain the model in more detail.

-

REVIEW #1

The authors present a follow-up to their 2017 study, where they modelled ellipsoid body (EB) and protocerebral bridge (PB) circuits in Drosophila, which have been shown to be involved in navigation and spatial orientation. In that study (which involved modelling spiking neurons in circuits deduced from recent connectome work), the same group proposed coupled symmetric and asymmetric circuits controlling orientation memory to a visual stimulus and feedback correction following body rotation (in the dark). That purely theoretical work made key predictions about how activity within these feedback circuits might govern spatial orientation. In the present work, the authors test some of these predictions by inhibiting or activating upstream inhibitory ring neurons. Crucially, they test this in freely walking flies, something that hasn’t been done before (all previous work was in tethered animals, complicating any assessment about self-motion and the contribution of ‘P-ring’ neurons towards correcting the position of the visual response in in the EB. The authors employ a version of Buridan’s paradigm, where flies walk back and forth between two visual stimuli, which they test in three phases: no stimulus, stimuli, and visual memory. Achieving consistent behavioral results for their inhibition/activation experiments (on two sets of neurons, c105-Gal4 or EIP-ring and VT5404-Gal4 or P-ring), they return to their 2017 model to observe their predictions are borne out. They are, quite admirably so. I have little to comment on the modelling work in the latter part of the manuscript, this seems to follow logically from their previous work, and the conclusions make sense.

This is a valuable contribution to this growing area of research, although a somewhat difficult paper to read. This is primarily because the average reader will not have read Su et al 2017 (I had to go back and read it carefully to understand this work), so without that background it is quite hard to follow. I have some suggestions on how the study could be made more accessible to the average reader, as well as some questions about the methods and results. These should be addressed before considering publication in eNeuro.

Major suggestions

1. Although references to Su et al 2017 are made, it would really help the paper if some schematic circuit model was presented up front, so that readers could appreciate where the manipulated EIP and P ring neurons reside in the EB/PB context. Why were these the most interesting neurons to manipulate in this study? Also, some better validation may be needed for why c105-Gal4 and VT5404 are the best drivers for these neurons (why were other Gal4s not tested to confirm, especially for VT5404 which seems more non-specific?).

Reply: Thanks for the reviewer’s suggestion, we have added a new panel in Figure 1 to illustrate the basic idea of the Su et al., 2017 model. We also revised text in Methods (line 285-309) to explain the basic ideas and the mechanisms of the model in a clearer way. Based on the model hypothesis, the upstream ring neurons control the activation of the individual model components that stabilize or update the head-direction encoding activity bump. Therefore, it is scientifically interesting to manipulate these upstream control neurons to see how the manipulation alters the behavioral performance in a spatial orientation task and whether the observations consist with the model predictions.

We have also redrawn Figure 5 to better illustrate the mechanisms of the model and the model predictions on the ring neuron manipulation.

As for the best GAL4 drivers. In addition to the two we used, there are quite a few others drivers expressed in the ring neurons we studied. For example, R31A12-GAL4 (Omoto et al., 2018) and VT39763-GAL4 (Lin et al., 2013) for EIP-ring neurons (R1 ring neurons), VT005404-GAL4 and R014G09-GAL4 (Omoto et al., 2018) for P-ring neurons (ExR4 ring neurons), and VT011965-GAL4 (Omoto et al., 2018) for C-ring neurons (R6 ring neurons). However, we have inspected the expression patterns of these drivers and most of them also expressed in many other neurons. The two drivers we used (c105-GAL4 for EIP-ring neurons and VT5404-GAL4 for P-ring neurons) are relatively cleaner than others. Since we performed behavioral tasks which involved perception, memory, decision and the movement, the specificity of a driver is crucial to the study. We have stated the reason of our driver choice in line 122-132 in the manuscript.

2. Any locomotion towards a stimulus after it is turned off is viewed as visual memory. Surely, flies have some walking inertia and will keep on walking straight irrespective of whether a visual was there or not. The authors support their view that this is memory by finding a significant correlation between prior stimulus duration and ongoing fixation after the stimulus disappears. They demonstrate 40s fixation duration for a stimulus that was on only 30s, and 80s fixation for a stimulus that was on for 2 minutes. That seems long, and I question their fixation metrics (the example in Figure 1 D/E certainly do not look like the tracks of a fixating fly. This may have to do with the ∼17% threshold for fixation ‘anywhere’ as opposed to the actual two objects? This needs better explanation. Also it is unclear why the pre-stimulus ‘fixation durations’ increase with stimulus duration, in Figure 1F. There is no stimulus in the pre-stage, so this makes little sense and this first result may confuse the average reader.

Reply: Yes. The long fixation duration is partially due to our definition (the 16.67% threshold) of fixation. Based on the definition, a fly can fixate at multiple angular quantiles during a unit time window (15 s) because the fly can move toward multiple directions with the percentages all larger than 16.67%. So, even when the flies performed random walk in the first stage, we still saw the flies exhibited long, but roughly equal fixation duration at every direction (Extended Data Fig. 1C). Therefore, our analysis and the performance measurements do not rely on the absolute duration of fixation in the third (post-stimulus) stage, but on the difference between the third and the first (pre-stimulus) stages. The larger the difference the more likely the flies maintain memory about the landmark locations. We rewrote some text in the Methods section (line 189-201) to explain this issue. Regarding the original Figure 1 D/E (Figure 1E/F in the revised version), the trajectories do not look like fixating on the landmark locations because the real fixation behavior is never perfect and is always mixed with other movements with various degrees, including random movement and movement along the rim. Therefore, comparing the movement between the third and the first stages is crucial for our study.

Finally, we agree that the fixation duration curve for the first stage in the original Figure 1F is confusion. The actual duration of fixation in the first stage does not increase with the length of the second and third stages. What we showed here is a “scaled” duration. This is because the first stage has a fixed length but the third stage has variable lengths. Therefore, we need to scale the values if we need to compare the fixation duration between the two stages on an equal basis. For example, to compare the fixation duration of a first stage of 90 s to a third stages of 120 s, we multiply the fixation duration of the first stage by 120/90 = 1.33. To avoid the confusion, now we use “fixation density” instead of fixation duration in Figure 1G. Fixation density is the fixation duration divided by the length of the corresponding stage.

3. There are multiple different fixation readouts: PI, percentage, and the ‘radar plots’. The radar plots seem most informative as they indicate angle deviation from the targets. However, the way they are shown it appears as if their polarity matters (left vs right of the targets), but it probably does not, it’s just an angle deviation. This may also confuse readers. Additionally, conclusions are made using these radar plots when it is unclear if significance was tested (e.g., line 395). Together, it is unclear why there are many different ways of assessing fixation.

Reply: The movement of fruit flies in the arena is quite complex and there is no single measurement that can fully describe all the characteristics of the movement patterns. Therefore, we used two levels of measurements. At the basic level, we use the PI (performance index) which is easily calculated and is an indicator of the spatial orientation memory, i.e. fixation at the 0{degree sign}/180{degree sign} directions. This single-valued metrics can be plotted and statistically compared easily. At the more advanced level, we use the radar plot to show fixation toward any angular quantile. The radar plot can tell the readers whether the strongest fixation is deviated from the 0{degree sign}/180{degree sign} directions. The radar plot is measured by two quantities: the fixation strength and the deviation angle. The fixation strength indicates the strength of the fixation at certain directions, not necessarily the 0{degree sign}/180{degree sign} directions. Therefore, PI and the radar plot serves different purposes and they are all useful in interpreting the data of our study. We have added detailed explanation on the reasoning behind the two measures (line 239-246).

In the original manuscript, we also showed fixation raster plots and fixation percentage plots (original Figure 4B, 4D, 4F & 4H) for individual flies. We agree that this yet another measure is a bit too complex and is not very useful for helping readers with understanding the main message of this figure. Therefore, we moved the plots to Extended Data Figure 1-3, 4-2, 4-3 and only used the radar plots and PIs in the revised Figure 4. The fixation raster plots and fixation percentage plots are good ways to show the raw behavior of individual flies, while performance index and radar plot are too noisy at the single fly level and are suitable for showing averaged behavior from a group.

Regarding the significance level of a radar plot, it is determined by the value of the Fixation Strength (FS) calculated from the plot. A radar plot indicates a significant fixation behavior if the FS is larger than 0.11. This number is determined by a statistical test. Briefly speaking, we analyzed the FS for all flies in the control groups in the pre-stimulus stage. The average FS was 0.0544 and the standard deviation was 0.0273. We took two standard deviations above the mean (FS=0.1090) as our statistical criterion for the fixation behavior. This statistical test is described in the Methods (line 235-238).

4. In Figure 4, a ‘percentage’ version of fixation is employed. This seems to be continuous measure based on the ∼17% threshold based on 6 (?) sectors (anything above 17% being non-random). This metric is hard to appreciate, as it is not clear how a fly can be determined to be fixating or not fixating from one moment to the next just by this threshold. Fixation seems to require time to reveal itself as such, and isn’t an instantaneous readout (at least for a fly in this kind of assay). Extended Although Figure 1-2 provides a level of explanation for how these ongoing fixation measures are determined, this metric remains problematic because the conclusions from Figure 4 simply sum this as a binary readout to propose that activation of both circuits during the memory phase abolishes fixation. From the data in Figure 4 D&H it does not look like fixation was abolished. It is hard to reconcile the conclusions with what can be seen on the graphs, and this may have to do with lack of clarity on what is meant by fixation duration and how it is calculated.

Reply: We agree with the reviewer and have modified Figure 4. In the new Figure 4 we only display the radar plots and PIs (performance index). The radar plots and PIs are measures taken over a long period of time and are can therefore reflect the true fixation behavior.

5. Chrimson activation during the third experimental stage abolishes fixation, but activation during the stimulus does nothing. Could this be simply because in the absence of the visual stimulus the red light is distracting, rather than it having an actual circuit-level effect? Was control experiment performed for non-ATR fed flies to control for the effect of red light, with and without the visual stimulus?

Reply: We thank the reviewer for the suggestion. We have performed additional control experiments on non-ATR fed flies and the results show that the red light does not abolish fixation behavior in these flies. The result is shown in Extended Data Figures 4-1, 4-2 and 4-3.

6. Although the authors made a good effort at using conditional approaches for suppression (Gal80), these remain chronic manipulations compared to optogenetics, so it is possible that long-term changes in circuit activity do not quite reflect what is being dynamically modelled. This could be mentioned in the discussion.

Reply: We agree with the reviewer. We wished to find a good optogenetic tool for transiently inhibiting the target neurons. We tried UAS-NpHR and used yellow light to hyperpolarize the neurons. But it did not work well because NpHR’s peak activation wavelength is visible to the flies. The onset of the activation light seriously disrupts the fixation pattern of wild-type flies in control experiments. We have added discussion about the possible issues of the chronic suppression in Discussion section (line 618-629).

7. The authors admit in the discussion that the ‘bump’ in the EB isn’t retinotopic, while their model requires a level of retinotopy. How a ‘random’ starting point for the bump might be implemented in their model could be discussed, as this seems a weakness in the model that does not reflect what has been observed.

Reply: The core assumptions of the model do not involve a fixed retinotopic mapping. We map each neuron to a fixed azimuthal position in the simulation is simply for the sake of coding simplicity. But we fully agree with the reviewer that the variable retinotopic mapping is an extremely interesting issue. The ‘random’ starting point of the bump is not difficult to be implemented in our model. We only need to send a uniform excitation to all EIP neurons to reset the system in the beginning of a trial. This excitation is going to elicit strong competition between the EIP neurons through the global inhibition of the ring neurons. The winner-take-all dynamics ensures that only a small set of neurons at a random location win the competition and this become the starting point of the bump. We aware that some studies suggested that the random starting point is induced by non-uniform inhibition from some of the ring neurons and the synaptic plasticity is involved. We will explore these different mechanisms in our next version of the model. We have included the discussion above in the Discussion section (line 630-637).

Minor points and corrections

Language in the abstract needs work, e.g. the 3rd sentence.

Line 199: switches

Line 361: was

Line 430: suppression ‘of’

Line 442: close

Line 446: Figure 4J does not seem to me called out.

Line 498: ‘that how’

Line 509: Should 6B be 6C?

Line 513: Should 6C be 6D?

Line 514: Delete 6D?

Line 580: Please explain what you mean by the ‘brief interruption’ of fixation and a ‘startle’ effect. This is not evident from the figures.

Line 590: When a fly stops fixating...

Line 591: Perhaps provide a reference or two for when you suggest a fly loses motivation or attention. Do flies even have attention?

Figure 1F: There is room on the figure to clarify what you mean by ‘scaled’. This seems important because it is not at all clear why a fly would be fixating if there are no visuals.

Extended Data 2-1: Please explain why there is loss of fixation at 15-30s specifically, in B&C.

Reply: We thank the reviewer for pointing out the grammar errors, incorrect citing and unclear descriptions. We have corrected all of them and carefully proof read the entire manuscript.

As for the ‘brief interruption’ of fixation and a ‘startle’ effect, these are from our earlier analysis showing that most flies tended to stop briefly after the sudden disappearance of the landmarks in the third stage but resumed the movement in a few seconds. This might be the reason why earlier studies only claimed a few seconds of memory if their analyses did not include the resumed movement. We originally included plots showing this phenomenon in an earlier version of the manuscript, but removed the plots later because we felt that they are not related to the main message of this paper. The removal made some text in the Discussion section awkward as the reviewer pointed out here. We have written and shortened this part of discussion section in the revision (line 610-617). We also dropped the word “startle” because it is an objective description.

Regarding “motivation or attention,” although motivation (studies by Waddell for example) and attention-like process (studies by Swinderen for example) in fruit flies have been investigated, we agree that we should be more careful when using these words. Therefore, we dropped “motivation or attention” and changed the sentence to “When a fly stops fixating, it is not clear whether the fly forgets the landmark directions or simply enters a different behavior state (but still remembers the landmark directions).”

For Figure 1G in the revised version, we now show the fixation density (fixation duration divided by length of the stage) instead of the fixation duration and the trend became clearer. Regarding why there is fixation during the first stage when no landmark is presented at all, this is because a fly often moves in a straight line toward a random direction for a significant amount of time (> 16.7% of a 15 s time window). This type of movement is registered as “fixation” in our definition. But if we look at the fixation durations for all directions, they are roughly equal in the first stage, showing no directional preference. We have presented the data in Extended Data Figure 1-2C.

As for the Extended Data Figure 2-1B and C, the reasons for the apparent loss of fixation are probably due to the brief pause resulted from the sudden disappearance of the targets as we discussed earlier. We observed that many flies stopped the fixation movement a few seconds after the offset of the landmarks. They may begin to move in random directions. But most of them resumed the fixation movement later. Due to the diversity of fly’s behavior, such phenomenon was strong in some flies but weak in others. The phenomenon was also observed in wild-type flies, not specific to flies carrying c105 > Kir2.1 or c105 > TNT. The loss of fixation between 15 s and 30 s shown here might reflect this phenomenon and it happened to be slightly stronger in these two group of flies. The data are not enough to tell whether this phenomenon is specifically stronger in flies with c105 > Kir2.1 or c105 > TNT. We would also like to emphasize that the fixation was not completely lost. The fixation strength (FS) was just slightly lower than the significance level we defined (line 235-238).

-

REVIEW #2

This paper reports some well-executed experiments to block or activate specific neurons in the CX of Drosophila during a free-walking short-term memory task. The results are of general interest but the interpretation seems overly focused on one specific model, without appropriate consideration of alternatives. This wider context would need to be addressed to make the paper acceptable.

Major issues:

The paper focusses on the ellipsoid body activity bump as a phenomenon that tracks the heading of the animal relative to the world, but neglects the likely function of this bump, i.e., to control moment by moment steering towards a current or remembered goal. Existing models [e.g. Stone et al. 2017] have indeed demonstrated that the circuitry is consistent with a steering mechanism, so it seems very strange that this paper models the actual behavior of the animal as a Markov chain of straight walks and random rotations.

This carries over into the interpretation of the compass circuit itself, in the assumption that there must be separate, alternatively activated circuits for holding the bump steady (for forward walking) and for moving the bump (during rotation). The ring neurons investigated are then assumed to be acting as gating the activity of one or other of these ‘contradictory’ subcircuits. But again, existing models [e.g. Kakaria et al. 2017, Pisokas et al. 2020] show that it is possible for the compass circuit to encompass both properties without gating, that is, to have sufficient recurrent feedback for bump stability, including in the absence of input, but sufficient sensitivity to input to alter the bump position, by appropriate parameter tuning.

The interpretation of ring neuron function also seems somewhat inconsistent with current evidence (although it is true the function is not fully understood). Specifically, the evidence [e.g. Kim et al. 2019, Fisher et al. 2020] would seem to suggest that ring neurons carry information about directional sensory cues (from vision, wind and possibly other modalities) and thus contribute to the location of the bump, possibly through adaptable synaptic connections, rather than just acting as general inhibition.

In summary, there remains enough uncertainty about this circuit that the authors are welcome to propose their own interpretation, and argue for support in their data, but this needs to be defended in relation to alternative models and potentially contradictory evidence, not presented as though it is the only solution.

Reply: We thank the reviewer for the suggestion. Indeed, there are several central complex models being proposed in the past few years. Each model has its own hypotheses and assumptions, and aims to elucidate different aspects of the neuronal mechanisms underlying central complex functions. We fully agree that it is important to compare our model with others and let the readers know whether there is any contradiction between them. We have added a concise version of the argument in the Introduction and Discussion sections (line 46-59 & 649-654) and provide a more detailed version here as follows.

Stone et al 2017: The model proposed in this paper covers almost the entire central complex of the bee brain, including PB, CBU (fan-shape body), CBL (ellipsoid body), LAL and NO. The model uses a simpler firing rate model instead of a more realistic spiking neuron model. This allows the authors to get rid of many complex issues associated with spiking neuron models, e.g. spike time variability, instability due to fluctuation of synaptic currents, etc. Therefore, they can construct a large scale model much easier. The model focuses on the path integration and the steering mechanisms, but keep the ellipsoid body component simple with minimal details. Their purpose is to model the robust homing behavior of bees and they are likely to have sophisticated neural circuits for this function. Fruit flies, by contrast, do not exhibite homing behavior, but their spatial orientation is well studied. Therefore, our model focuses only on the EB-PB circuit and do not model the steering circuit. The steering part is only handled by a mathematical behavior model, or more specifically, a Markov-chain model. We do not think Stone’s steering circuit model can be used in fruit fly directly, but developing an extended central complex model covering FB, NO and LAL is indeed our long-term goal.

Kakaria et al. 2017, Pisokas et al. 2020: Both studies have two distinct differences from ours. 1. The models mainly focus on the stability of the bump when stimulus is presented and when the stimulus is off. They did not investigate how a bump can be shifted according to the body rotation during darkness. Our model, in contrast, addresses the bump shifting mechanism when the landmark is off. The bump is particularly fragile during this period, as also shown in these two studies. Our model shows that the gating mechanism is required if you want to shift the bump during this period, otherwise the fragile bump will easily collapse. This does not contradict to Kakaria et al. 2017 and Pisokas et al. 2020 as they did not address this function. 2. They assumed that the main inhibition source is from the intrinsic neurons of the protocerebral bridge (PB). We agree that the PB intrinsic neurons can be a potential source of inhibition in addition to the ring neurons. In our earlier model development, we tried to use PB intrinsic neurons alone and found that the bump is less stable than if we use ring neurons alone. So this is why our model goes with the ring neurons. But the differential level of stability can be due to the parameter tuning or the choice of neuron/synapse models. We will address this issue in future model development.

Kim et al. 2019, Fisher et al. 2020: Indeed, these and a few other studies suggested that ring neurons are characterized by specific visual receptive fields and may be responsible for formation of the bump and their shifting in accordance to moving stimuli. However, we did a careful comparison based on the morphologies and soma locations using fluorescence and EM images, and we concluded that these two papers investigated different types of ring neurons from us. Kim et al. 2019 mainly studied EPG (or EIP) wedge neurons but not specific ring neurons and Fisher et al. 2020 mainly studied EPG neurons and R2 & R4d ring neurons (part of A and O ring neurons in Lin et al., 2003) (Figure 3 and Extended Data Figure 5 in Fisher’s study). By contrast, our study investigated EIP ring neurons (R1 ring neurons) and P ring neurons (R6 ring neurons). On the one hand, we cannot rule out that the ring neurons investigated in our study also perform similar functions as studied in Kim et al. 2019 and Fisher et al. 2020, but on the other hand these two papers also used computational models to elucidate their theories and the most direct experimental evidence on the plasticity of GABAergic synapses and non-global inhibition is still lacking. Further studies are required to clarify these issues.

In addition, the actual presentation of the computational model is inadequate. How many units are simulated in the model? Using what kind of neural model? What is the exact connectivity it encapsulates? How do the ring neurons interface? If I understand correctly, the model does not actually model the behavior of the animal, such as the steering observed when visual stimuli are present. Yet the closed loop interaction of the circuit with behavioral control is key to understanding function. Indeed this is also quite critical to bridge their interpretation of the (possible) effects of the manipulations on the bump characteristics to the observed behavior of the animal.

As such, the final conclusions of the paper are not well supported. For example, it could be argued that all the effects demonstrated relate to the extent to which a stable bump can be maintained in the absence of external orienting stimuli, rather than to “crucial roles of two types of GABAergic ring neurons in spatial orientation working memory”. Their model does not include mechanisms of either memory or behavior, but only shows phenomenological effects on the bump following the removal of stimuli; not “a detailed picture of how the global inhibition (from the ring neurons) modulates the memory”.

Reply: We thank the reviewer’s comment. We have updated our Methods section by including a brief description of our model (line 285-309), including the neuron model (leaky integrate-and-fire), the synaptic dynamics (conductance based with exponential decay) and how different types of neurons interact. The synaptic weights are given in Table 1. However, due to the space limit, we refer to the original paper (Su et al. 2017) for the full details of the model. Regarding the behavior model, we do not model the neural circuits that generate the steering behavior as it is going to involve too many neuropils with insufficient data required for building a spiking neural network model. However, we still model the behavior using statistical approach as described in line 311-340 and in Extended Data Figure 6-1. Our behavior model is described by a Markov-chain process in which a fruit fly switches between the rotation and forward-movement states stochastically with probabilities inferred from the behavioral analysis. This model is consistent with our observation (Extended Data Figure 6-1). We would like to argue that including a closed-loop steering mechanism in the model is not necessary for the present study because the model is only used to explain how manipulating different ring neurons affects the presence of the bump or the accuracy of the bump position. The effects on the bump is not related to how downstream steering circuits orient the fly. We simply assume that if the bump disappeared, or if the bump is deviated from the head-direction, the downstream steering circuits cannot accurately orient the fly toward the correct direction. This assumption is sufficient to link the circuit model with the observed behavior and is also the standard approach in many neural circuit models of memory. However, we fully agree that it is important to include a steering circuit model in the future if we would like to study correlation between spatial memory and navigation.

Regarding our conclusion on the role of the ring neurons on spatial orientation working memory, we agree that the statement should be toned down. The conclusion of the present study should be stated separately for the experiment and the model part. First, our behavioral experiments indeed demonstrated the loss of memory (at the behavioral level) about the positions of the landmarks after manipulation of the ring neurons. Therefore, the ring neurons indeed play roles in spatial orientation working memory. Second, our modeling study showed that by simulating the neuronal manipulation as in the experiments, the bump stability and accuracy changes. Third, these changes were consistent with the observed behavioral deficits, suggesting that the ring neurons affect the performance of the spatial orientation memory task through their regulatory roles in the activity bump. Using the term “consistent with” we mean the model provides one explanation for the observed behavior deficits. We cannot rule out other explanations such as altered steering mechanism, which is not included in the model. We have rewritten the last paragraph of the Discussion section to help the readers to correctly interpret our study (lines 668-674).

Minor issues:

There are a number of odd citations (possibly something has become confused?) E.g. line 34, these seem an odd choice of citations for ‘neuromorphic engineering’ and at line 36, Dewar et al. seems inappropriate.

Reply: We thank the reviewer for pointing out this problem. We have carefully examined all citations and corrected inappropriate ones.

Lines 45-47, there are additional models of the CX that should be cited.

Reply: We thank the reviewer for the suggestion. We have cited more models of the central complex in line 46-55.

Lines 55-56, the underlying theory of attractor circuits (which should be cited) has long been able to account for both stability and updating of the bump - there is no need to conceive of them as contradictory mechanisms.

Reply: Indeed, the theory of attractor networks has long been able to account for both stability and updating of the bump. But most models used firing rate or continuous models, and implementing those ideas with spiking neuron models is a lot more difficult because the spiking neuron models are much more dynamically unstable than the firing rate models. There were only a few spiking neuron models of the head-direction circuits but they either built based on the classical attractor network (not the EB-PB circuit), or did not address the questions we asked here. But we agree with the reviewer that we should not present the stability/update as contradictory mechanisms and we have removed this part of text.

Line 65-70 It is unclear how the prediction of the Su et al model was in any way specific or how it was verified by the study of Turner-Evans et al.

Reply: We agree that this is not an appropriate claim and have removed this sentence.

Line 212 it seems misleading to call this the ’simulated behavioral task’ when the control of behavior is not simulated. I became quite confused at this point in the manuscript, assuming the simulated fly must be somehow directed towards the stimulus or the bump memory, and only understanding on line 500 in the results that this is not the case.

Reply: We use the term “simulated behavioral task” is because we still implemented a simplest form of behavioral model, the Markov-chain model, which models the random switch between two behavioral states: forward movement and rotation. Interestingly, although during the stimulus and post-stimulus stages the flies move toward the landmark locations with a probability higher than the chance, our analysis showed that the behavior during these two stages still match the Markov-chain statistics. The only difference between random movement in the pre-stimulus stage and the movement in the stimulus/post-stimulus stages is that out of many behavioral state switches, there were a few rotations toward the landmark locations. In fact, in the early phase of the present study, we did implement an additional state in which the flies rotate toward the landmark locations. However, including this state did not affect the outcome of the model nor change the conclusion of the study. Moreover, adding this state induced some control issues that is related to the downstream steering mechanism, which is out of the focus of the present study. Therefore, we decided to keep the model simple. We have revised the text in the Methods section (line 311-339) to better explain the rationale behind the behavior model.

Line 266-267, the definition of fixation duration seems extremely permissive, that is, counted as any time (in a 15s sliding window) the fly movement direction is between [-30,30] or [150,210] degrees more than the pure chance level of 1/6 of the time. As such it is maybe not so surprising that they see ‘unexpectedly long” duration of the behavior? From Fig 1, it appears flies spend a substantial amount of their time (around 1/3) ‘fixating’ during the pre-stimulus stage, by this measure. It is not clear in the later discussion (line 523 on) if the measure used here is actually comparable to that used in previous studies.

Reply: Indeed, the definition of the fixation is permissive. But we stress that the fixation here is just a description of a behavioral pattern, and we do not claim that the absolute value of the fixation duration indicate the presence of memory. The true presence of memory is indicated by the difference in the fixation duration (at 0{degree sign}/180{degree sign}) between the pre-stimulus and post-stimulus stages. Please check the revised Figure 1G in which we plot the fixation density (fixation duration/duration of stage) for the pre-stimulus stage, post-stimulus stage and their difference, which is about 20% when the stimulus stage was 120 s long. This means that on average, the flies spent 20% more time fixating on the cued locations in the post-stimulus stage than in the pre-stimulus stage. If we multiple this number by the duration of the post-stimulus stage (120 s), we obtain an estimated memory duration about 24 s. This number represents the change of the fixation behavior evoked by the earlier exposure to the stimulus, and hence indicates the trace of memory. We add the above estimate in the Result section (line 399-410). In order to prevent the readers from mistaking the absolute fixation duration for the memory duration, we replot Figure 1G by showing fixation density and the difference between the pre- and post-stimulus stages instead of the fixation duration. Moreover, we have tone down the statement about “unexpectedly long” memory in Discussion (lines 610-611).

Line 375 “While the flies with suppressed EIP-ring neurons exhibited the same PI as did the wild-type flies during the stimulus stage (Fig. 2B), the PI of the flies with suppressed EIP-ring neurons decreased significantly during the post-stimulus stage and was markedly lower than that of the wild-type flies (Fig. 2C).” How is this possible when PI is defined (line 269) as the difference between the third and first stage? I assume the PI can also be difference between second and third stage, but this needs to be made clear in the methods.

Reply: We thank the PI for spotting this issue. Yes, PI for the second (or third stage) is defined as the difference between the second (or third) and the first stage. We have corrected the definition in the Methods section (Eq 1, now in line 201).

Line 478 on, “the EP-PB model is only a neural circuit model and does not include any behavioral component. Nevertheless, we can still infer the behavioral outcomes...” This speculation would be much more convincing if a behavioral component was included in the model, to demonstrate that the changes in bump activity (e.g. broader width, greater drift) actually do result in the behavioral effects that were observed.

Reply: As we have argued in our responses to the reviewer’s earlier questions, the present model includes a simple behavioral model which meets the purpose of the present study. Structural, functional and behavioral data that are required to construct a detailed circuit-level behavioral model was not available at the time of the development of the present model. Although the detailed connectome data for the central complex have recently published, it takes a few years to analyze the data and construct a new model. The purpose of the present model is only to demonstrate that under various neuronal manipulations, whether the bump is still available and whether the bump location accurately reflects the head-direction. We agree that including a detailed behavioral model allows us to reproduce more experimental observation such as the complex walking patterns. This is indeed our plan for future study.

Line 521 on, it seems surprising in the model results that the ‘photoactivation’ causes the bump to be permanently abolished (see also fig 6), i.e., without recovery after the additional inhibition is removed. In most ring attractor models, a bump will form spontaneously (in the absence of input that influences the bump position).

Reply: Yes. We aware that a bump would spontaneously generated in the absence of input. In fact, adding a mechanism to generate a spontaneous bump is quite easy in our model. We simply need to add a global excitation to the whole population of EIP neurons. After the original head-direction-indicating bump disappeared due to the ring neuron photoactivation in the third stage, the winner-take-all dynamics will ensure that one neuron at a random location wins the competition and forms a new spontaneous bump. Because the new bump forms at a random location, the fruit flies lose the reference to the location of the landmarks (which are disappeared already). So adding a spontaneous bump or not both lead to the same conclusion: the flies fail to fixating on the previously cued locations. Therefore, adding this mechanism or not does not change the conclusion of the model. We are not saying that the mechanism underlying the spontaneous bump formation is not important. It is important if we want to model detailed movement patterns of flies in the future. We have revised some text (line 637-648) to clarify this issue.

Line 535 “when the P-ring neurons were activated in the last 30 s of the stimulus stage, the activity bump was lost in the subsequent post-stimulus stage in a small percentage (29 out of 120) of the trials”. What factor in the simulation causes this non-deterministic result? How was the proportion affected by the tuning of the parameter representing photoactivation (line 246)?

Reply: The main factor causing the bump loss is the intrinsic instability in the P-ring circuit. In our model, the P-ring circuit is responsible for shifting the bump and is intrinsically less stable than the C-ring circuit, which has perfect recurrent excitation that maintains the bump in the same location. When the P-ring neurons were photoactivated, the P circuit was suppressed continuously. This is not a problem during the stimulus stage as the bump simply follows the stimulus and no additional shifting mechanism is required. However, this becomes a problem if the fruit fly happened to be in the rotation state when the photoactivation ends and the post-stimulus stage starts. The P-ring circuit takes time (a few tens of milliseconds) to kick back in and to shift the bump. There is a certain probability that the bump collapses during this delay. Indeed, the bump losing probability can be affected by several model parameters such as the excitation strength of photoactivation, membrane and synapse time constants, etc. Therefore, we do not link this phenomenon to the observed behavior anymore in the revised manuscript. Accordingly, we removed some text in the Results and a figure panel (original Figure 6H right).

The grammar in the paper also needs some improvement, here are some of the errors noted:

Lines 5-6, this is ungrammatical: “However, how does this system involved in the orientation working memory at the behavioral level is not well understood.” -> “How this system is involved”.

Line 73 Problem with tense, it should be “in particular those involved in stabilizing and updating”.

Line 142 Problem with tense, it should be “to transiently activate EIP-ring”.

Line 361 grammar: “the third stage were longer than the that in the first stage", maybe change this to “the third stage was longer than that in the first stage”.

Line 430 grammar, maybe change it something like this “suppression of these downstream”.

Line 438 grammar: the word “interference” is not used properly.

Line 442 spelling should be “the flies were close to”.

Line 498: remove “that”.

Line 598 “in three aspects”. The following text lists four aspects.

Reply: We thank the reviewer’s for pointing out the grammar errors, we have carefully proof read the manuscript and modified all of the grammar errors.
