# Supplementary figures and images for: Coordination through Inhibition: Control of Stabilizing and Updating Circuits in Spatial Orientation Working Memory
Source: eNeuro. 2021 Sep 7;8(5):ENEURO.0537-20.2021. doi: 10.1523/ENEURO.0537-20.2021 (PMC8425968; doi:10.1523/ENEURO.0537-20.2021)

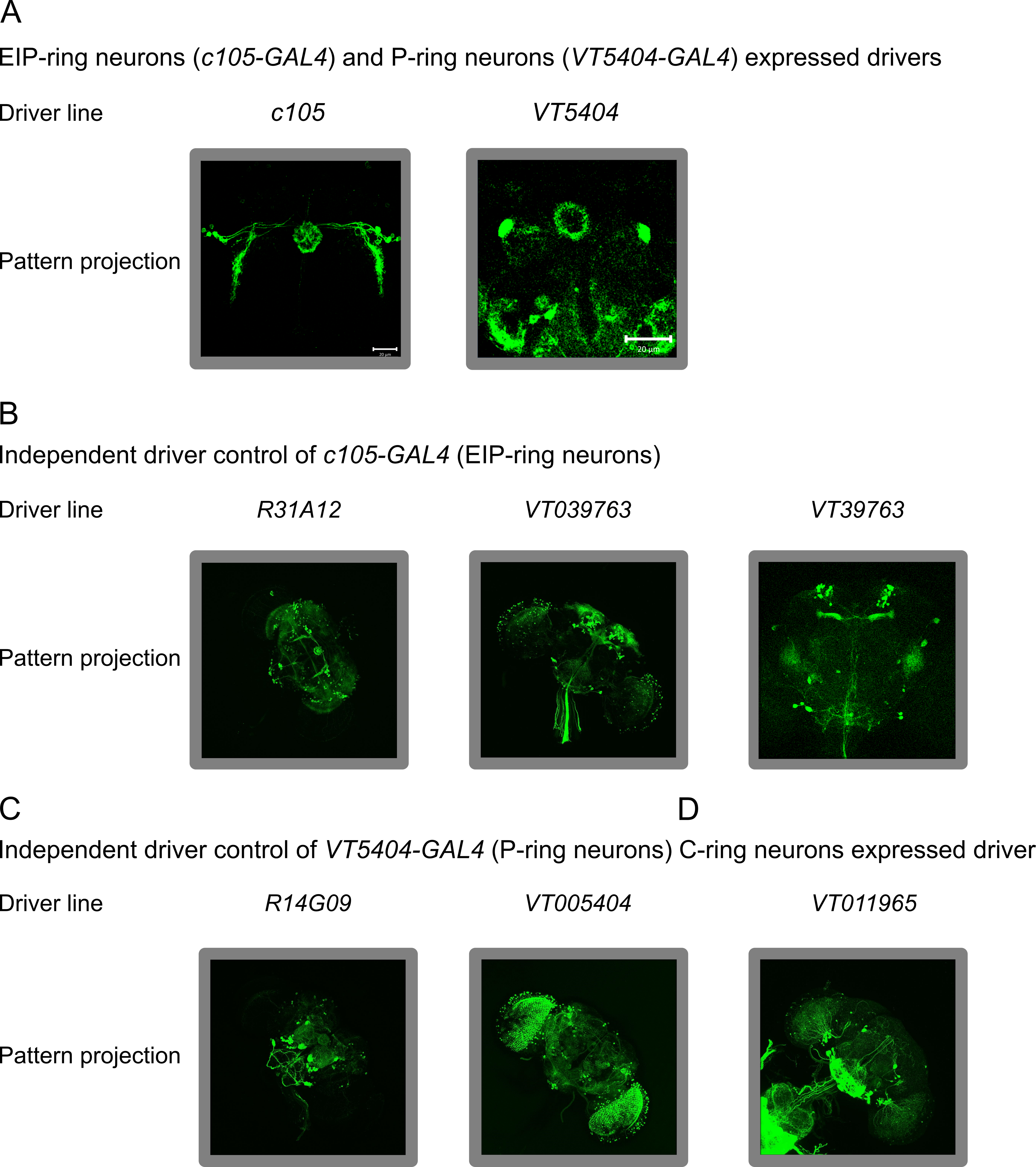

Supplement: Extended Data Figure 1-1 — The fluorescent images of the GAL4 drivers that are expressed in the EIP-ring neurons, P-ring neurons, or C-ring neurons. A, The EIP-ring neurons expressed driver c105-GAL4, and the P-ring neurons expressed driver VT5404-GAL4. B, The EIP-ring neurons expressed drivers R31A12-GAL4 (Jenett et al., 2012; the image is from Janelia Research Campus, https://flweb.janelia.org/cgi-bin/view_flew_imagery.cgi?line=R31A12#), VT039763-GAL4 (Tirian and Dickson, 2017; Janelia Research Campus, https://flweb.janelia.org/cgi-bin/view_flew_imagery.cgi?line=VT039763#), and VT39763-GAL4. C, The P-ring neuron expressed control drivers VT005404-GAL4 (Tirian and Dickson, 2017; Janelia Research Campus, https://flweb.janelia.org/cgi-bin/view_flew_imagery.cgi?line=VT005404#) and R14G09-GAL4 (Jenett et al., 2012; Janelia Research Campus, https://flweb.janelia.org/cgi-bin/view_flew_imagery.cgi?line=R14G09#). D, The C-ring neuron expressed driver VT011965-GAL4 (Tirian and Dickson, 2017; Janelia Research Campus, https://flweb.janelia.org/cgi-bin/view_flew_imagery.cgi?line=VT011965#). The Rubin lines R31A12-GAL4, R14G09-GAL4 (Jenett et al., 2012) and the VT lines VT039763-GAL4, VT005404-GAL4, VT011965-GAL4 (Tirian and Dickson, 2017) are from FlyLight database (https://www.janelia.org/project-team/flylight) and are provided under CC BY 4.0 license (https://reurl.cc/a5AD29). The drivers shown in B–D are less specific to the target neurons and are also expressed in many other brain regions. Therefore, they are not used in the present study. Download Figure 1-1, TIF file. [file enu-eN-NWR-0537-20-s07.tif]

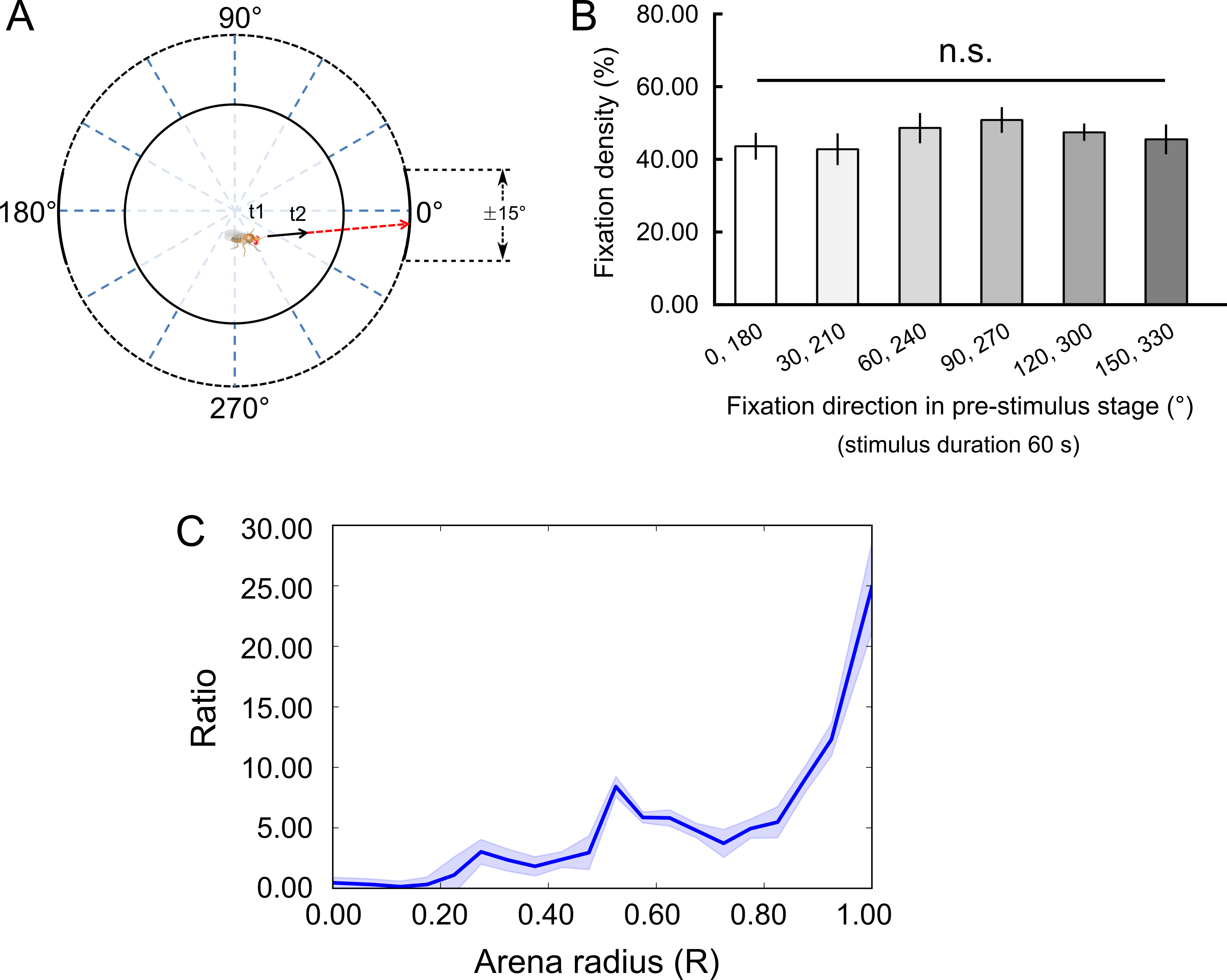

Supplement: Extended Data Figure 1-2 — The definition of movement direction and the trajectory distribution. A, In the behavioral task, we recorded the movement direction of the fly in each time step by calculating the vector (black arrow) that connects the positions of the fly in the previous (t1) and present (t2) time steps. The movement direction is defined by the projection (red arrow) of the vector on the LED screen. The movement direction is 356° (or –4°) in this case. B, The population-averaged fixation density (fixation duration/stage duration) toward each pair of quantiles in the prestimulus stage. The fixation density of each pair of quantiles are comparable, suggesting that the flies did not exhibit directional preference in the first stage. C, The distribution of the trajectories of flies on the platform along the radius during the prestimulus stage. The shaded area indicates the SEM. The values were normalized by the area spanned in each unit length of the radius. Download Figure 1-2, TIF file. [file enu-eN-NWR-0537-20-s08.tif]

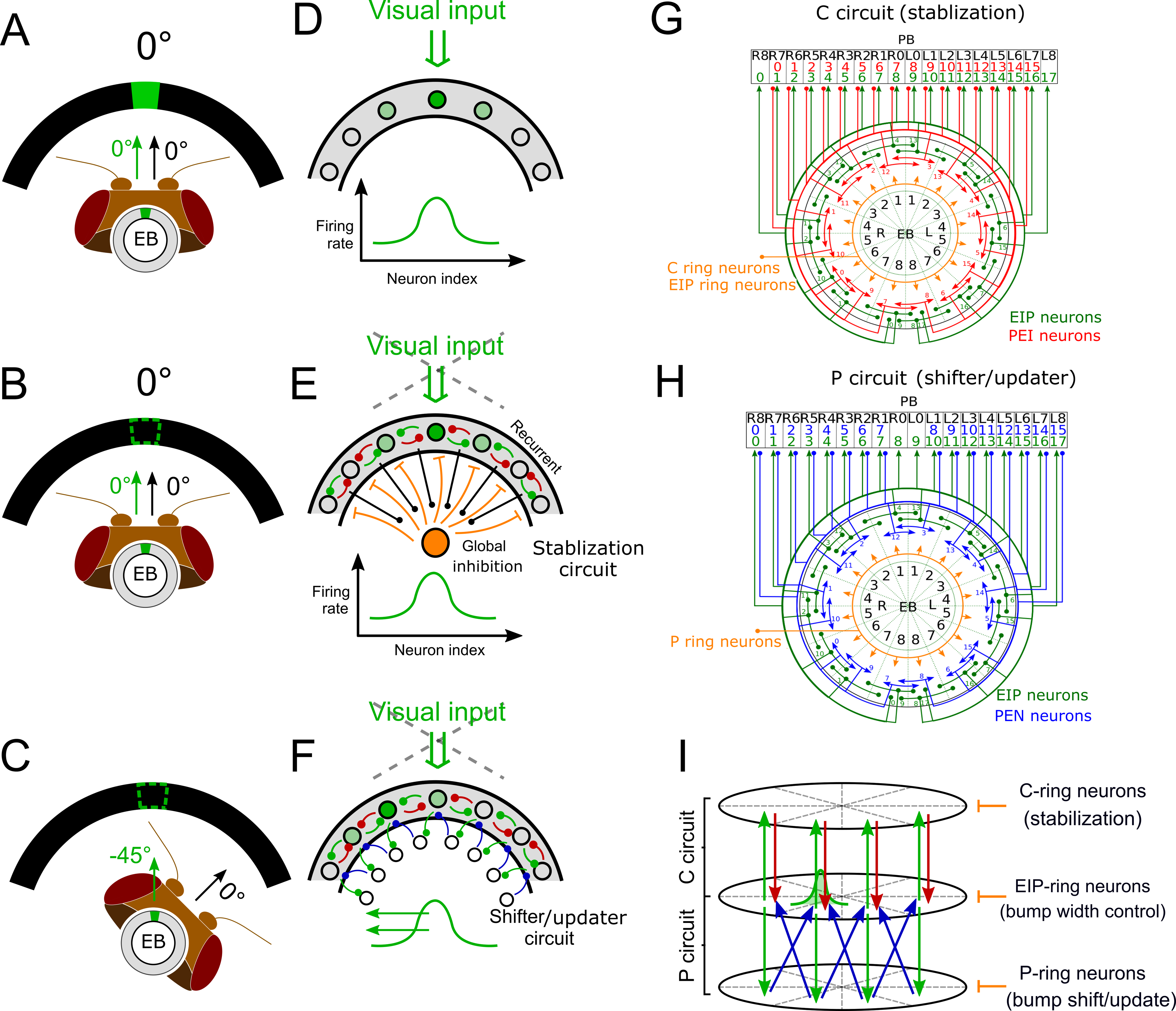

Supplement: Extended Data Figure 1-3 — Schematics of the fruit fly compass circuits and its model. A, Fruit fly EB (the grey torus) exhibits localized neural activity, termed activity bump (green), which corresponds to the direction of the visual cue on the screen. B, The activity bump persists when the visual cue is turned off. C, When the fruit fly changes its heading, the location of the activity bump shifts (or updates) so that it always indicates the head direction with respect to the cued direction. D, The activity bump can be simply generated by localized input to neurons in EB. E, However, according to the attractor network model, the persistency of the bump after offset of the visual cue requires two types of synaptic connections. First, locally recurrent excitation (green and red), which creates the reverberatory activity in the absence of input. Second, the global feedback inhibition (orange), which controls the width of the activity bump so that it does not spread throughout the whole population of neurons. We call these two sets of connections “stabilization circuit.” F, Moreover, the shift of bump location due to the change of heading can be achieved by a shifter (or updater) circuit which form counterclockwise (blue, as shown) or clockwise (not shown) feedforward excitation, which propagates the activity in either direction. G, H, The specific connections as described in panels E, F were discovered in neurons that interconnect EB and PB in recent connectomic studies. The recurrent excitatory circuits are formed by the EIP (green) and PEI (red) neurons (C circuit), while the shifter circuits are formed by the EIP and PEN (blue) neurons (P circuit). These neurons are modulated by several types of GABAergic ring neurons (orange). The Su et al. (2017) model suggested that the coordinated activation of the C and P circuits are crucial for the function of spatial orientation. I, To simplify the visualization of the circuits, we redraw them using a three-ring representation, which highlights the [file enu-eN-NWR-0537-20-s09.tif]

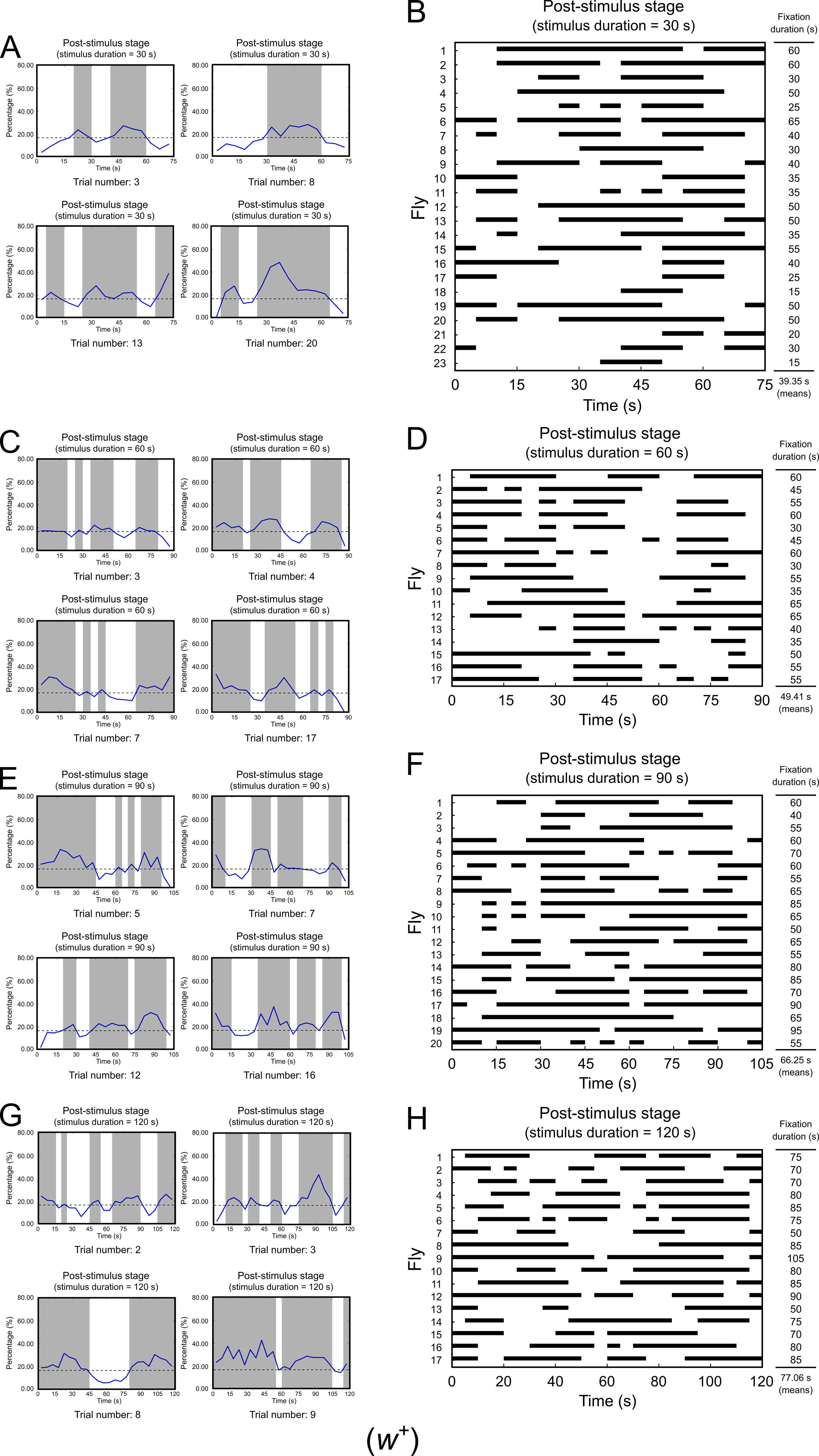

Supplement: Extended Data Figure 1-4 — The movement patterns of Individual flies for difference stimulus stage durations. A, Percentage of fixation for four example trials of wild-type flies. The shaded regions indicate the periods in which the percentage of movement toward the landmark positions is more than 16.67% (see Materials and Methods). B, Distribution of the fixation bouts (black bars) in the poststimulus stage for each wild-type flies (stimulus stage duration = 30 s). In this condition, the average fixation duration of each single trial is 39.35 s. C, D, Same in A, B, but with a stimulus duration of 60 s. In this condition, the average fixation duration of each single trial is 49.41 s. E, F, Same in A, B, but with a stimulus duration of 90 s. In this condition, the average fixation duration of each single trial is 66.25 s. G, H, Same in A, B, but with a stimulus duration of 120 s. In this condition, the average fixation duration of each single trial is 77.06 s. Download Figure 1-4, TIF file. [file enu-eN-NWR-0537-20-s19.tif]

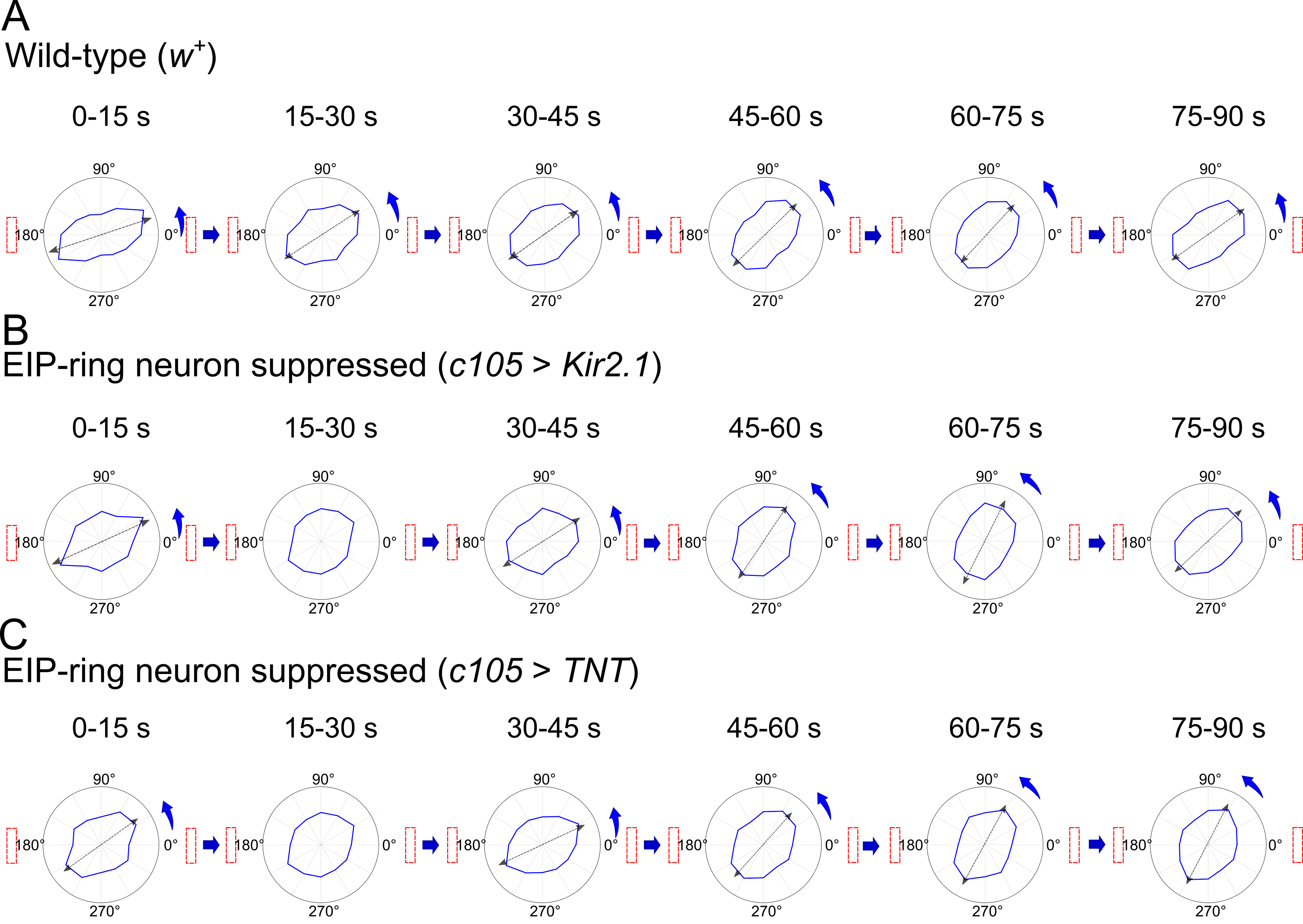

Supplement: Extended Data Figure 2-1 — Radar plots of the flies in the control groups (wild type: w+) and flies with suppressed EIP-ring neurons. A, Wild-type Drosophila (genotype: w+). B, C, Flies with suppressed EIP-ring neurons (B for 32°C, c105-GAL4, tub-GAL80ts;; UAS-Kir2.1 and C for 32°C, c105-GAL4, tub-GAL80ts;; UAS-TNT) in the poststimulus stage. Download Figure 2-1, TIF file. [file enu-eN-NWR-0537-20-s10.tif]

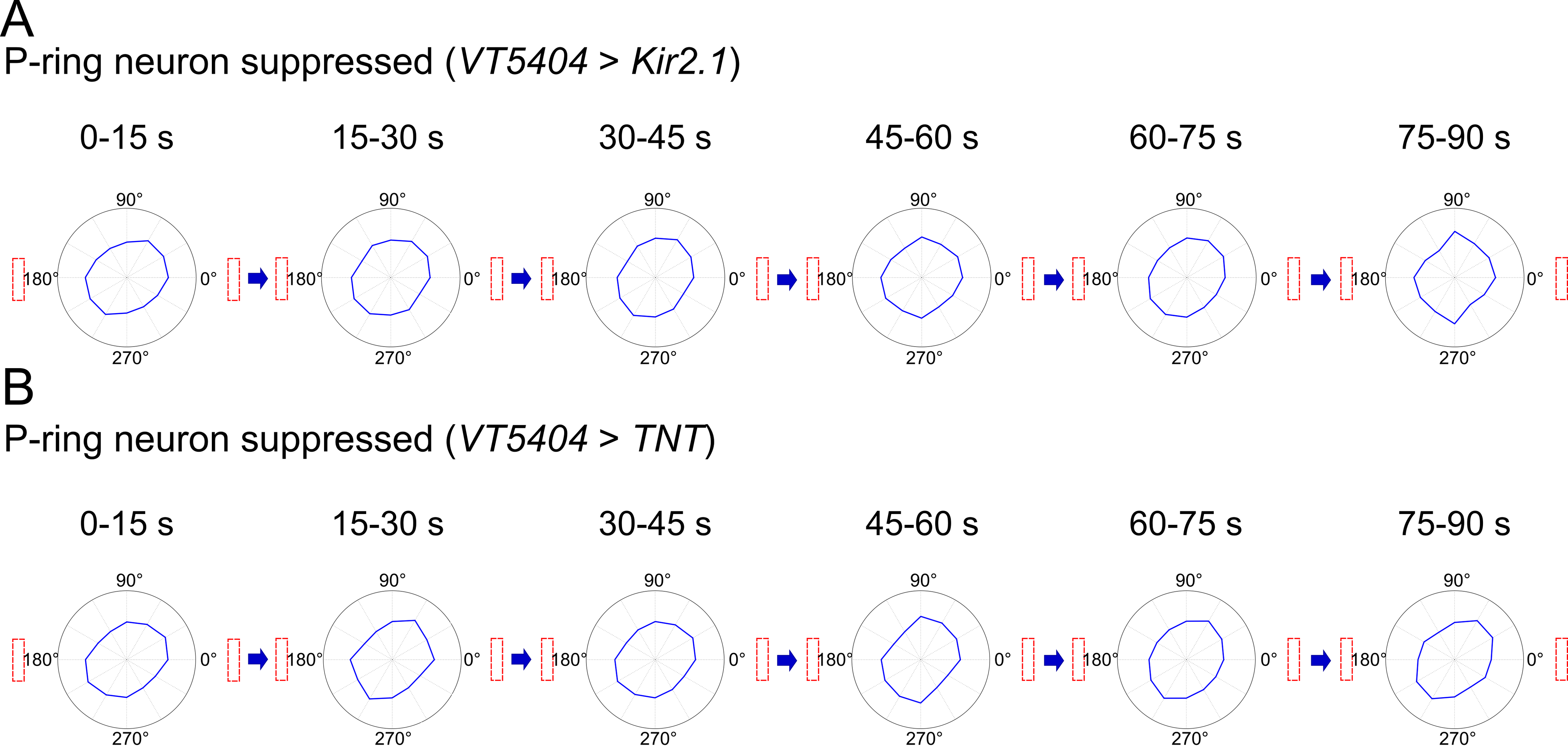

Supplement: Extended Data Figure 3-1 — Radar plots of flies with suppressed P-ring neurons in the poststimulus stage. A, 32°C, ;;VT5404-GAL4, tub-GAL80ts/UAS-Kir2.1. B, 32°C, ;;VT5404-GAL4, tub-GAL80ts/UAS-TNT. Download Figure 3-1, TIF file. [file enu-eN-NWR-0537-20-s11.tif]

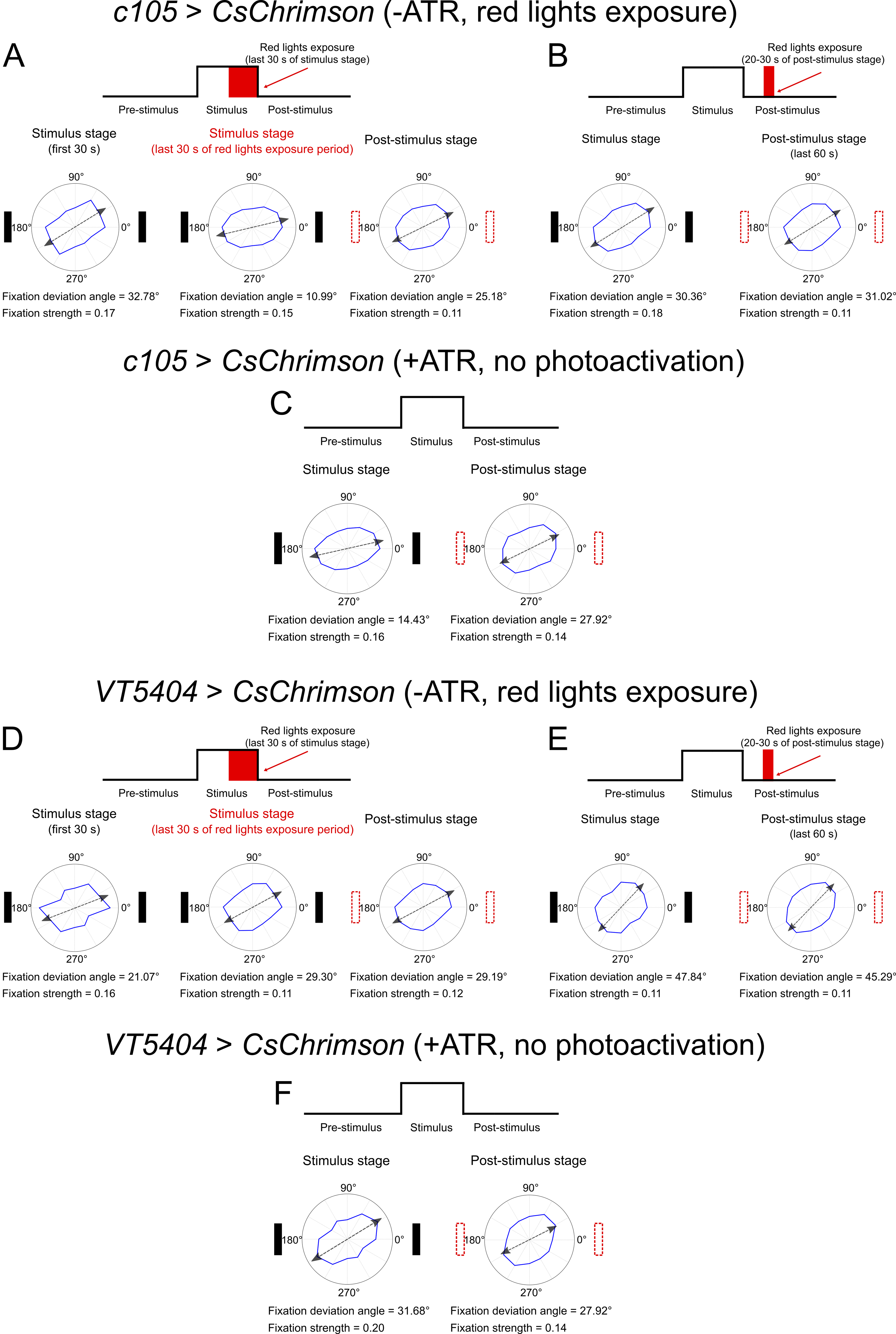

Supplement: Extended Data Figure 4-1 — Performance of control groups with the same types of transgenic flies used in the optogenetic experiments. A, B, Performance for flies that carry c105-GAL4;;UAS-CsChrimson.mVenus, but without feeding ATR. The schematic protocol (top) and the radar plots for the stimulus stage (bottom left, first 30 s; bottom middle, last 30 s) and the poststimulus stage (bottom right). B, Same as in A, but with photoactivation during 20–30 s of the poststimulus stage. C, Same as in A, but with ATR fed and no photoactivation. D–F, Same as in A–C, but for flies that carry ;;VT5404-GAL4/UAS-CsChrimson.mVenus. Both control groups exhibited a similar fixation performance to that of the wild-type flies. Download Figure 4-1, TIF file. [file enu-eN-NWR-0537-20-s12.tif]

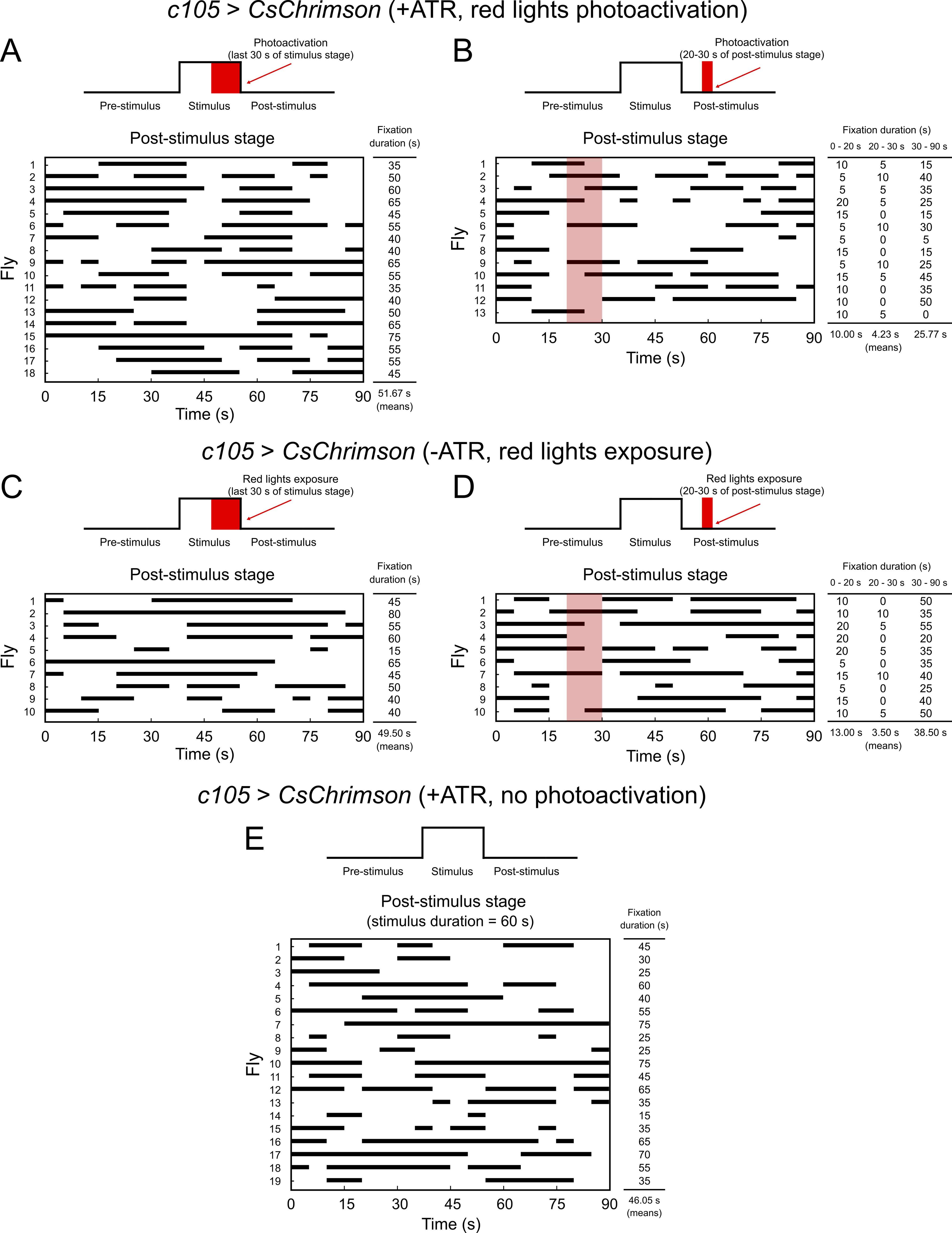

Supplement: Extended Data Figure 4-2 — Movement patterns of individual flies in the photoactivation experiments. A, Distribution of the fixation bouts (black bars) of each fly with the photoactivation of the EIP-ring neurons (c105-GAL4;;UAS-CsChrimson.mVenus) during the last 30 s of the stimulus stage. B, Same as in A, but with photoactivation during 20–30 s of the poststimulus stage. C, D, Same as in A, B, but for non-ATR fed. E, Same as in C, but for ATR fed and no photoactivation was delivered. Download Figure 4-2, TIF file. [file enu-eN-NWR-0537-20-s13.tif]

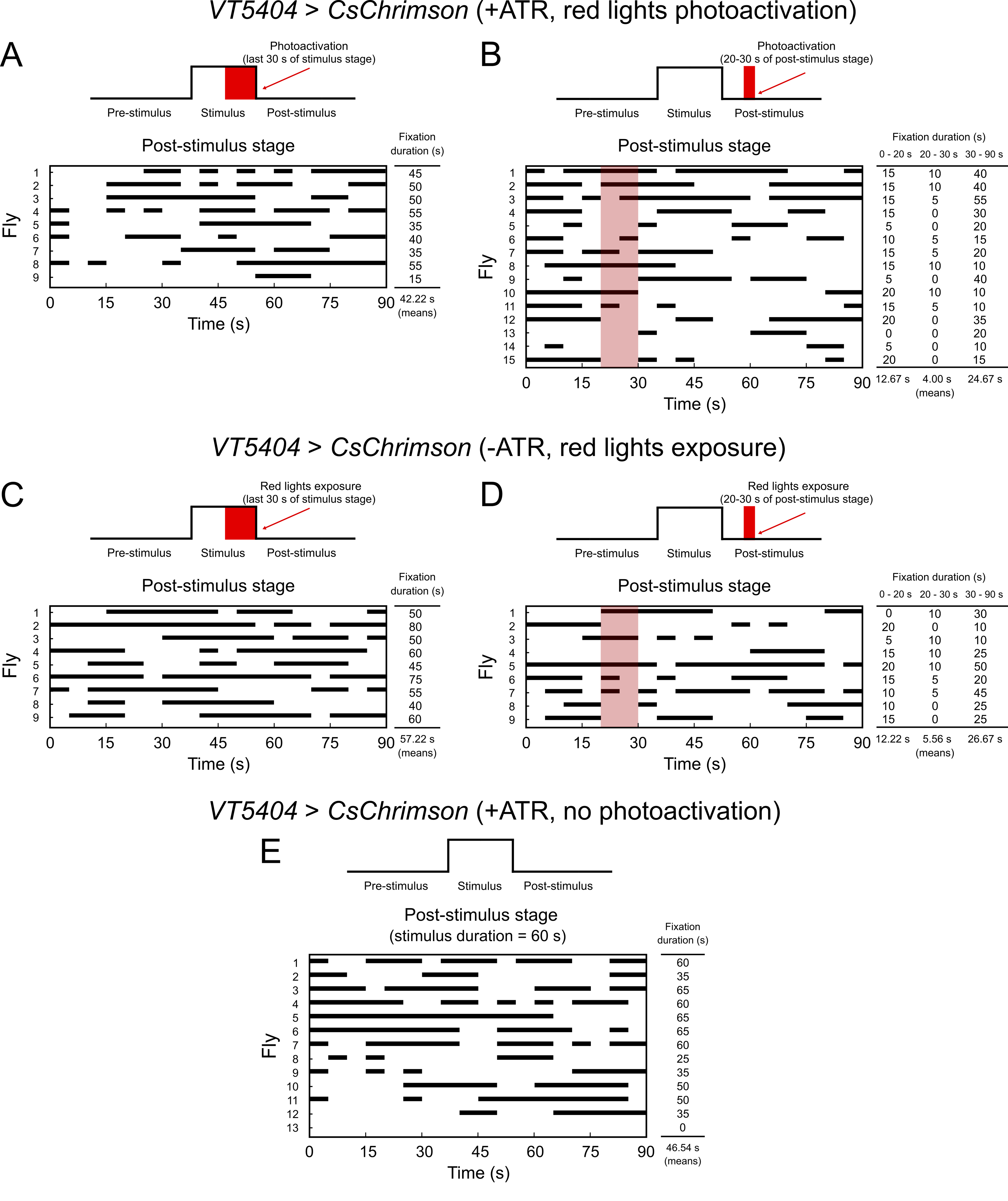

Supplement: Extended Data Figure 4-3 — Same as in Extended Data Figure 4-2, but for flies that carry ;;VT5404-GAL4/UAS-CsChrimson.mVenus. Download Figure 4-3, TIF file. [file enu-eN-NWR-0537-20-s14.tif]

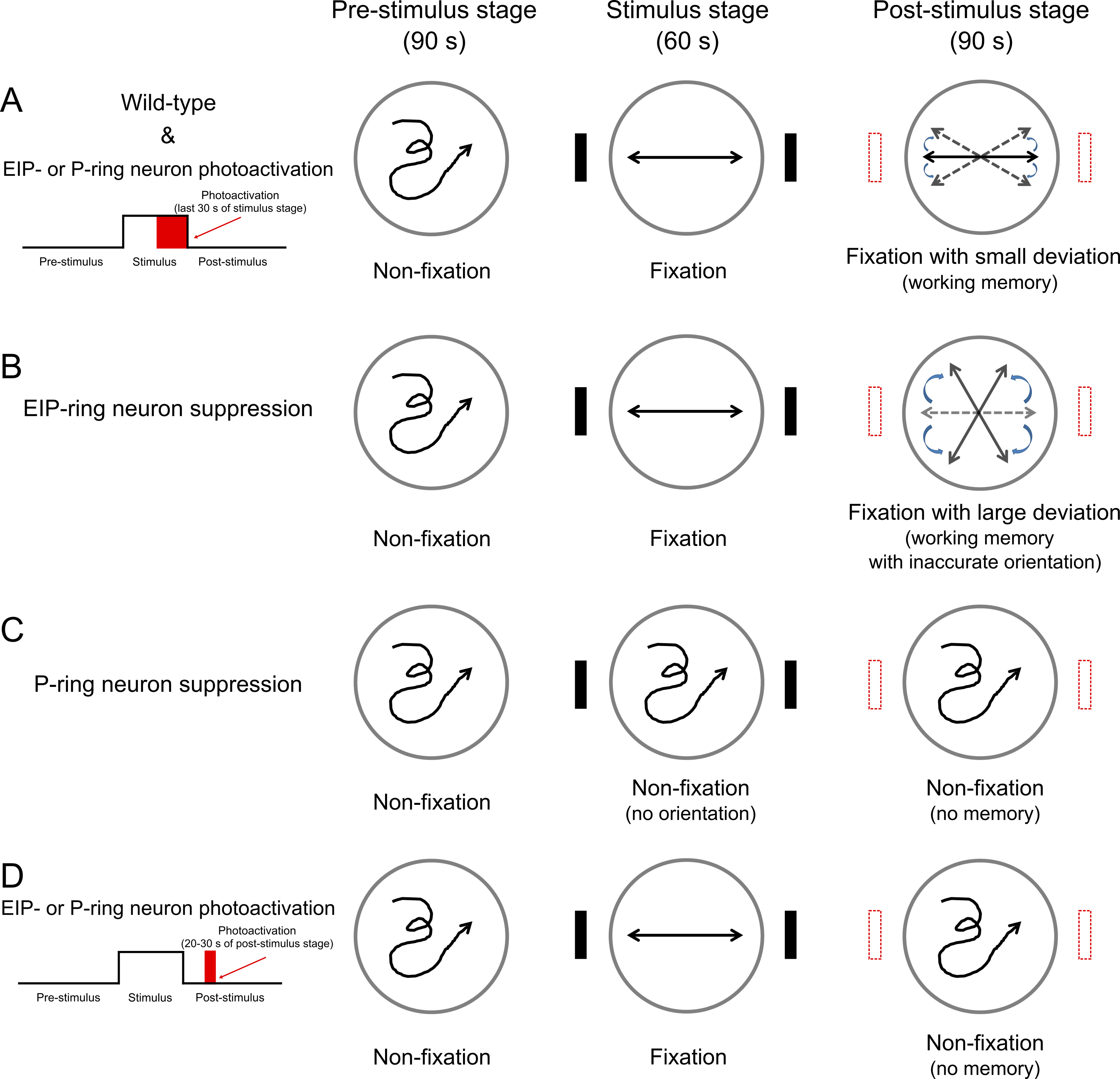

Supplement: Extended Data Figure 5-1 — Schematic that summarizes the behavioral effects of EIP-ring and P-ring neuron suppression and photoactivation. A, Wild-type flies maintain fixation behavior in both the stimulus stage and the poststimulus stage, in which the flies exhibited a slight deviation in the fixation direction. Flies with transient photoactivation of the EIP-ring or P-ring neurons during the stimulus stage exhibited a similar behavioral pattern. B, Flies with suppressed EIP-ring neurons exhibited fixation behavior with large deviation in the poststimulus stages, respectively. C, Flies with suppressed P-ring neurons could not maintain fixation behavior, indicating loss of spatial orientation. D, Flies with transient photoactivation of the EIP-ring or P-ring neurons during the early poststimulus stage eliminated the fixation behavior. Download Figure 5-1, TIF file. [file enu-eN-NWR-0537-20-s15.tif]

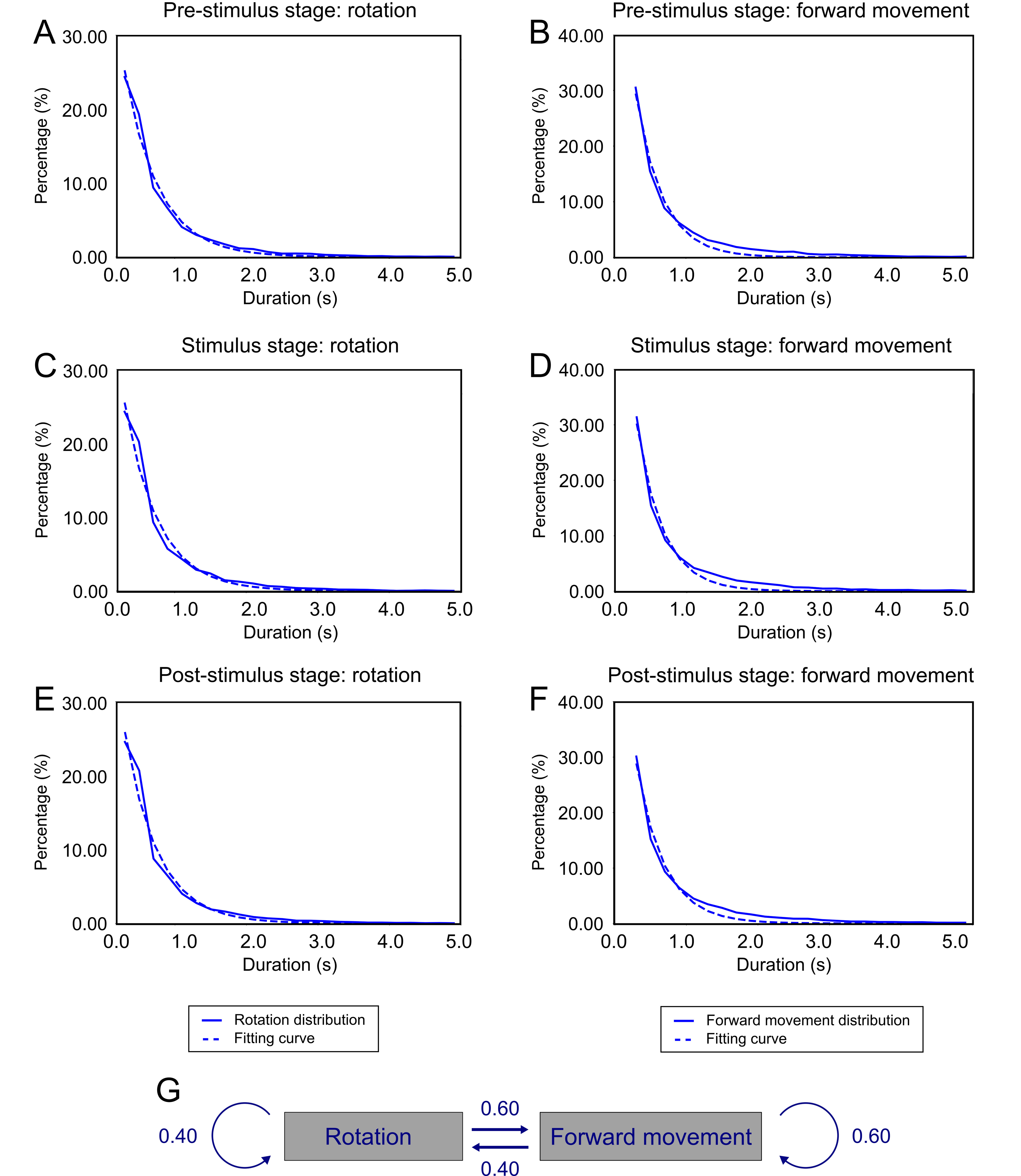

Supplement: Extended Data Figure 6-1 — Distributions of duration of different behavioral states and the Markov-chain model of behavioral control. Dashed lines represent the fitting curves, which is given by y=α*βx0.3. α is the fitting parameter and represents the probability of staying in the same state in each time window of 0.3 s. The exponential shape of the distribution of state duration indicates the Markov-chain dynamics. A, The distribution of duration of the rotation state in prestimulus stage. α = 0.473, β = 0.533, and R2 = 0.986. B, Same as in A but for the forward movement state, α = 0.657, β = 0.448, and R2 = 0.985. C, The distribution of duration of the rotation state in stimulus stage. α = 0.482, β = 0.529, and R2 = 0.978. D, Same as in C but for the forward movement state, α = 0.688, β = 0.439, and R2 = 0.984. E, The distribution of duration of the rotation state in poststimulus stage. α = 0.495, β = 0.523, and R2 = 0.975. F, Same as in E but for the forward movement state, α = 0.621, β = 0.464, and R2 = 0.984. G, The behavioral model was constructed based on the Markov-chain dynamics and described the probability (given by the numbers next to the arrows) of a fly switching between behavioral states. Download Figure 6-1, TIF file. [file enu-eN-NWR-0537-20-s17.tif]
